# What Makes a Good Protein–Protein Interaction Stabilizer: Analysis and Application of the Dual-Binding Mechanism

**DOI:** 10.1021/acscentsci.3c00003

**Published:** 2023-04-14

**Authors:** Shu-Yu Chen, Martin Zacharias

**Affiliations:** Center for Functional Protein Assemblies, Technical University of Munich, 85748 Garching, Germany

## Abstract

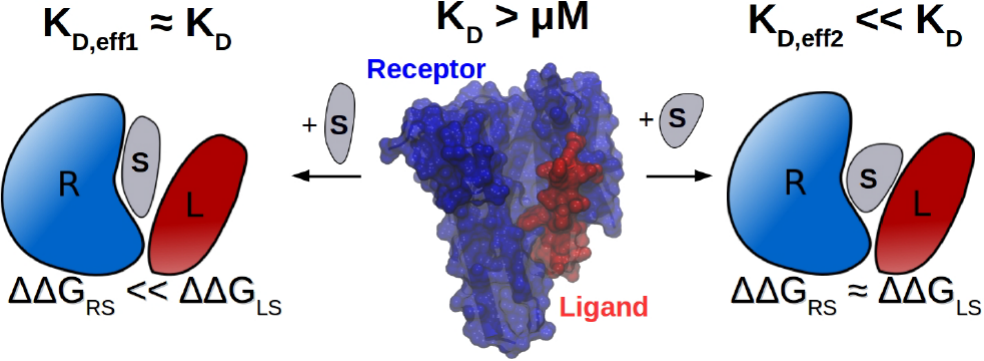

Protein–protein interactions (PPIs) are essential for biological processes including immune reactions and diseases. Inhibition of PPIs by drug-like compounds is a common basis for therapeutic approaches. In many cases the flat interface of PP complexes prevents discovery of specific compound binding to cavities on one partner and PPI inhibition. However, frequently new pockets are formed at the PP interface that allow accommodation of stabilizers which is often as desirable as inhibition but a much less explored alternative strategy. Herein, we employ molecular dynamics simulations and pocket detection to investigate 18 known stabilizers and associated PP complexes. For most cases, we find that a dual-binding mechanism, a similar stabilizer interaction strength with each protein partner, is an important prerequisite for effective stabilization. A few stabilizers follow an allosteric mechanism by stabilizing the protein bound structure and/or increase the PPI indirectly. On 226 protein–protein complexes, we find in >75% of the cases interface cavities suitable for binding of drug-like compounds. We propose a computational compound identification workflow that exploits new PP interface cavities and optimizes the dual-binding mechanism and apply it to 5 PP complexes. Our study demonstrates a great potential for *in silico* PPI stabilizers discovery with a wide range of therapeutic applications.

## Abstract 2

Simulation studies reveal potential drug binding cavities at several protein−protein interfaces. Principles for identifying specific stabilizers are elucidated, and a design work flow is developed.

## Introduction

Complex formation of proteins plays a fundamental role in the majority of biological processes such as cell fate,^1^ immune reactions, and signal transduction^2,3^ and is involved in many human diseases,^4,5^ offering substantial therapeutic potentials for modern drug design.^4,6,7^ Approaches to protein–protein interaction (PPI) modulation are typically inhibition^8^ but more recently also stabilization^9−11^ of interactions. PPIs can be inhibited by allosteric effects (involving binding of compounds not necessarily at the interface) or by targeting the protein–protein interface directly such that protein partners can not form a complex. However, the design of PPI inhibitors suffers from the general feature of a typically flat and large buried interface area (>1500 Å^12,13^^12,13^) and also often hydrophobic^13^ surface, which is frequently considered as “undruggable” for small molecules.^14,15^ Larger peptide-based inhibitors^16^ can be adopted as a promising strategy with low toxicity, and indeed several peptides have been approved as clinical drugs.^17^ In particular, cyclic peptides with predefined conformation and good cell permeability have received increasing attention in PPI inhibitor design,^18−20^ and using *in silico* “hot-spots” matching with cyclic peptides has been proposed for rapid rational design.^21^

For many diseases, promotion or stabilization can be more helpful than the inhibition of PPIs. Such stabilization can be achieved by small organic molecules through an allosteric (not at the interface) or a direct interface binding mechanism.^11^ The former mechanism describes the allosteric regulation of one protein partner upon stabilizer binding, which increases the binding affinity to another binding partner, while the latter mechanism describes the direct binding of the stabilizer at the PPI interface with a potentially druggable cavity for small molecules.^22,23^ With several advantages compared to PPI inhibitors, stabilization of PPIs has become an emerging field in recent years. Zarzycka et al.^24^ and Andrei et al.^10^ surveyed the available protein–protein-stabilizer complexes and discussed different groups of stabilizers according to the stabilized PPI type and the origin of the stabilizers, respectively.

So far, only a few drug design efforts have been directed toward stabilizer design. For example, fragment-based drug discovery was used for the stabilizer design of the adapter protein 14-3-3 with p65-derived protein,^25^ the tumor suppressor protein p53,^26^ oncogenic transcription factors TAZ,^26^ and estrogen receptor-derived peptide.^27^ Sijbesma et al. performed structure-based virtual screening over nearly 6 million compounds from the Molport database and tested 13 compounds *in vitro*, among which 2 are validated as potent stabilizers.^28^ Tang et al. combined molecular dynamics (MD) and a molecular docking technique to investigate 22 14-3-3 protein-peptide-stabilizer complexes and concluded that a simulation time of 20–50 ns combined with molecular mechanics Poisson–Boltzmann surface area (MM/PBSA) and generalized-Born surface area (MM/GBSA) methods are useful to identify stabilizers among decoys.^29^

With the rapid advance in protein structure prediction,^30,31^ protein–protein docking,^32−34^ and protein complex structure prediction,^35^ the prediction of potential PPI stabilizers from merely the knowledge of just protein sequences can be a promising route at relatively low cost. To achieve such a goal, it is of fundamental importance to understand the functional mechanisms of the existing stabilizers.

**Figure 1:**
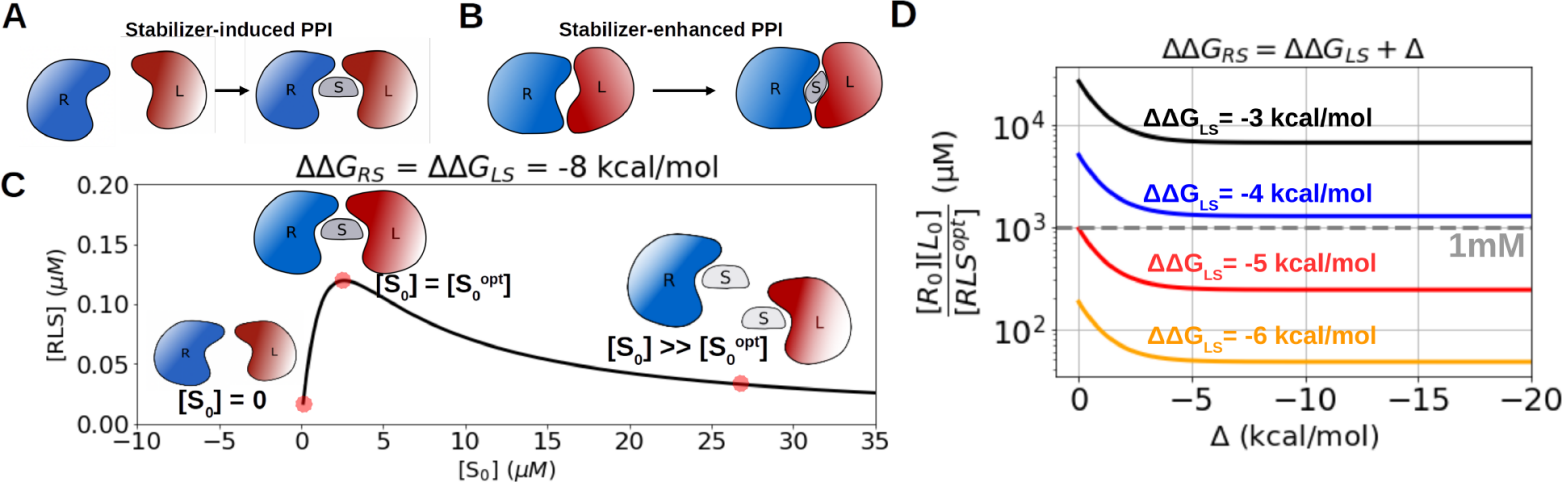
Theoretical model of the dual binding mechanism. (A) Schematics of stabilizer-induced PPI where two noninteracting or weakly interacting proteins R and L are stabilized by the presence of a stabilizer S. (B) Schematics of stabilizer-enhanced PPI where the interaction between a native RL complex is enhanced by the presence of a stabilizer S. (C) Schematics of the theoretical model according to eq 1. [R_0_] and [L_0_] are set to 1 μM, and the cooperative factor ϕ is set to 1 (noncooperative binding). Schematics from left to right represent the ternary complexation with no stabilizer, optimal stabilizer, and oversaturated stabilizer. (D) The theoretical dependency of the effective dissociation constant *K*_RL,eff_ = [*R*_0_][*L*_0_]/ [RLS^opt^] with different sets of (ΔΔ*G*_RS_, ΔΔ*G*_LS_). The gray dashed line shows the boundary between the millimolar and micromolar ranges of *K*_RL,eff_.

In the first part of this study, we discuss the theory of PPI stabilization and emphasize the importance of a dual-binding mechanism. Next, we analyze existing protein–protein (PP) complexes with bound stabilizers using MD simulations combined with the calculation of interaction free energies between protein partners and the stabilizer. We use the term stabilizer-induced PPI for complexes that do not form without a stabilizer (Figure 1A), whereas stabilizer-enhanced PPI includes those that form complexes already without stabilizers (Figure 1B). We find that the more potent stabilizers in most cases distribute the calculated interaction evenly between both protein partners, regardless of the total interaction free energy between the PP complex and the stabilizer. This follows the expectation based on a mathematical model for the equilibrium binding state, whereas less potent stabilizers tend to bind more strongly to one protein than the other. However, a few compounds mediate the stabilization through an allosteric mechanism by indirectly increasing the PP affinity (4 out of 18 cases). In addition, indirect positive and negative (destabilization) effects on the direct protein partner interaction were observed.

Importantly, we show that 80% of the stabilizer-binding pockets can be detected *in silico* by direct computational probing of the PP complex (without stabilizer) crystal structure. Those pockets hidden in the PP complex structure can be revealed by running short MD simulations and subsequent pocket detection. We also demonstrate that the dual-binding mechanism can be useful to identify potential PPI stabilizers. Based on the model, we propose a protocol for PPI stabilizer discovery combining pocket probing, molecular docking, and MD simulation. We also check a large set of known PP complexes for druggable pockets and find, for 75% of the cases, interface cavities useful for stabilizer binding and apply the suggested protocol to a subset of 5 PP complexes. It indicates promising results that could be implemented in the structure-based drug design of PPI stabilizers.

## Results and Discussion

### Stabilization of PPI through the Dual-binding Mechanism

Arguably, the aim of a potent PPI stabilizer is to increase the level of formation of the RLS ternary complex. In the case where R and L do not interact, e.g., stabilizer-induced PPI, the system is similar to the bivalent scaffold discussed by Yang and Hlavacek^36^ (Figure 1A). With the total concentration of receptor [*R*_0_], ligand [*L*_0_], and stabilizer [*S*_0_], the formation of the RLS complex [RLS] at the equilibrium state can be formulated as
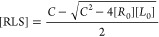
1where
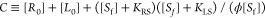
2ϕ is the cooperative factor, [*S*_f_] indicates the concentration of the unbound stabilizer, and *K*_RS_ and *K*_LS_ are the dissociation equilibrium constants of the RS and LS complex, respectively. Equation 1 describes that the RLS complex cannot be efficiently formed when [*S*_0_] is too high because of the saturation of the individual RS and LS complexes, and there exists an optimal concentration of the total stabilizer, [*S*_0_^opt^] = , that maximizes the RLS formation to [RLS^opt^] (Figure 1C).

With the relation *K*_D_ = *e*^ΔΔ*G*/(*RT*)^, where *K*_D_ is the dissociation equilibrium constant and ΔΔ*G* is the Gibbs binding free energy, *k*_B_ is Boltzmann constant, and *T* is the temperature, we can study how [RLS^opt^] changes with ΔΔ*G*_RS_ and ΔΔ*G*_LS_ according to eq 1. In the case where ΔΔ*G*_RS_ < ΔΔ*G*_LS_, e.g., the stabilizer binds more strongly to the receptor, enhancing both ΔΔ*G*_RS_ and ΔΔ*G*_LS_ by a small amount improves the effective dissociation equilibrium constant *K*_RL,eff_ = [*R*_0_][*L*_0_]/[RLS^opt^] much more efficiently than enhancing ΔΔ*G*_RS_ alone. This indicates that the protein that binds weaker to the stabilizer, namely, max {ΔΔ*G*_RS_, ΔΔ*G*_LS_, plays a more decisive role in determining the stabilization efficiency (Figure 1D). Hence, a dual-binding activity is effective for a stabilizer to efficiently induce the RLS complex formation. At the extreme case where ΔΔ*G*_RS_ is strong enough that all receptors are in the stabilizer-bound form, it is apparent that the formation of RLS depends solely on ligand-stabilizer interaction. Note, the dual-binding mechanism is one mechanism to stabilize PPI, and other allosteric mechanisms are discussed in the next paragraphs.

### Investigation of Known PP Complexes with Bound Stabilizer

**Figure 2:**
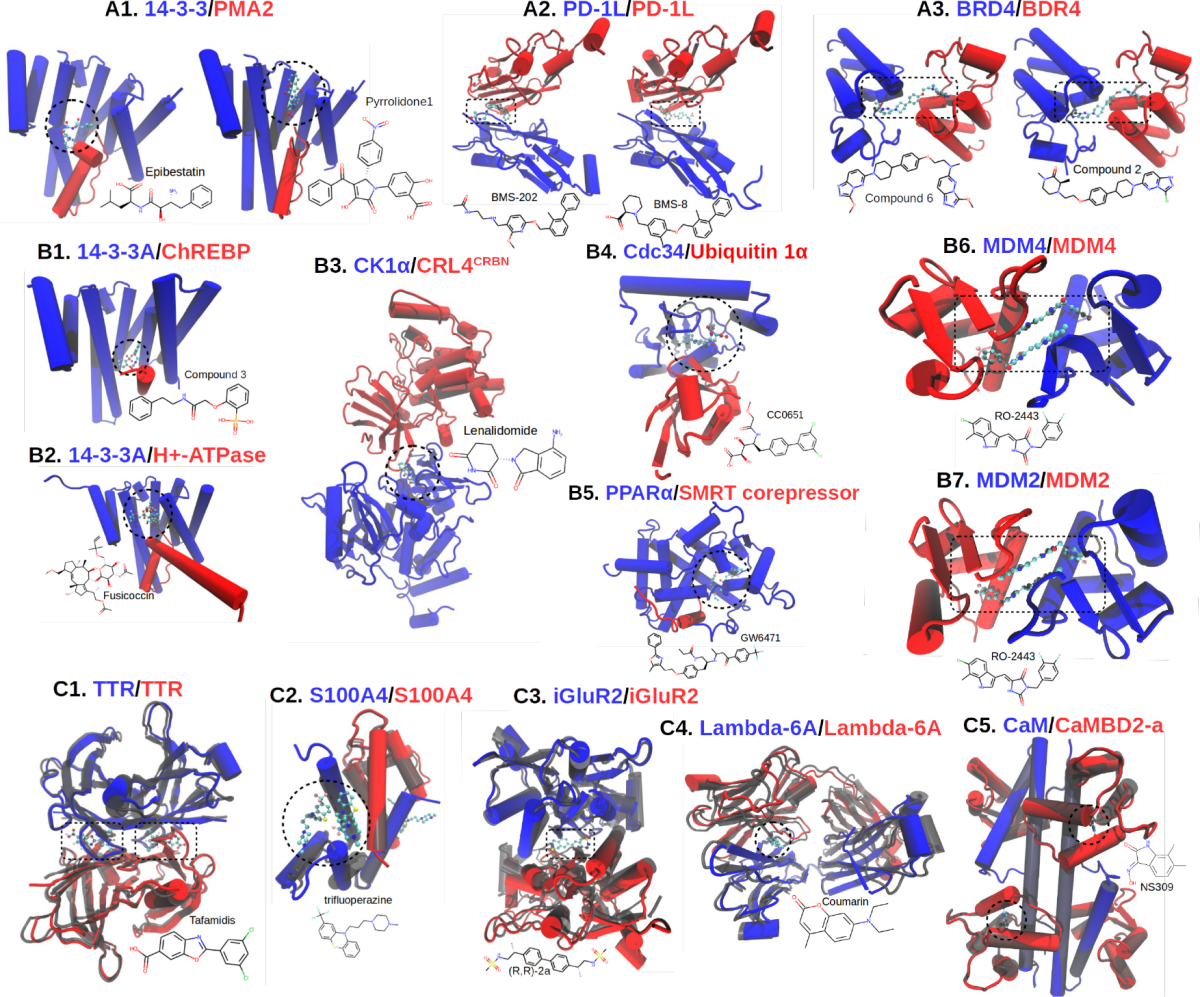
3D structures of the RLS ternary complexes investigated in this study. Receptor and ligand are labeled blue and red, respectively. The corresponding PDBIDs are listed in Table 1. The locations of stabilizers are encircled by black dashed lines. Set A (top row) contains 6 RLS complexes with common RL partners binding to a more potent stabilizer (left) and a less potent stabilizer (right). Set B (middle rows) contains 7 RLS complexes with only one stabilizer without an available RL structure. Set C (bottom row) contains 5 RLS complexes with the 3D structures of the RL complexes available (transparent gray).

In the present study, we investigate 18 stabilizers binding to 15 different PPIs using MD simulations and pocket detection techniques. An overview of our data set is given in Table 1 and visualized in Figure 2.

**Table 1:** Protein–Protein Complexes Stabilized by Bound Compounds Investigated in This Study^a^

	name (R/L)	PDBID (RLS)	stabilizer	PDBID (RL)	*TM-score^37,38^	R residue	L residue
Set A. Stabilizer-Induced PPI with 2 stabilizers							
A1	14-3-3/PMA2	*(a)**3m50*(39)	*Epibestatin*		0.9960	9-240	926-956
		*(b)*3m51(39)	Pyrrolidone1				
A2	PD-1L/PD-1L	(a) *5j89*(40)	*BMS-202*		0.9641	18-134	18-134
		(b) 5j8o(40)	BMS-8				
A3	BRD4/BRD4	*(a)**5ad3*(41)	*Compound 6*		0.9634	42-168	42-168
		(b) 5ad2(41)	Compound 2				
Set B. Other Stabilizer-Induced PPI							
B1	14-3-3/ChREBP	6ygj(28)	Compound 3			4-232	117-136
B2	14-3-3/H^+^-ATPase	2o98(42)	Fusicoccin			2-241	905-956
B3	CK1α/CRL4	5fqd(43)	Lenalidomide			47-436	14-303
B4	Cdc34/Ubiquitin 1α	4mdk(44)	CC0651			3-184	3-74
B5	PPARα/SMRT	1kkq(45)	GW6471			200-269	682-700
B6	MDM4/MDM4	3u15(46)	2x RO-2443			26-107	26-107
B7	MDM2/MDM2	3vbg(46)	2x RO-2443			26-109	26-109
Set C. Stabilizer-enhanced PPI							
C1	TTR/TTR	3tct(47)	2x Tafamidis	4tlt^48^	0.9912	11-125	11-125
C2	S100A4/S100A4	3ko0(49)	4x Trifluoperazine	3cga^50^	0.9331	2-94	2-94
C3	iGluR2/iGluR2	3bbr(51)	(R,R)-2a	3b6q^52^	0.7748	390-775	390-775
C4	lambda-6A/lambda-6A	6mg5(53)	Coumarin	6 mg4^53^	0.9867	1-214	1-214
C5	CaM/CaMBD2-a	4j9z(54)	2x NS309	4j9y^54^	0.9790	395-491	2-147

a Note: R residue and L residue indicate the residue number of the receptor protein and the ligand protein used in this study, respectively. *TM-scores in set A are calculated between the RL conformations between the two crystal structures, and TM-scores in set C are calculated between the RL conformations from the RL and RLS crystal structures. For set A italic font indicates the complex with the more potent stabilizer.

**Figure 3:**
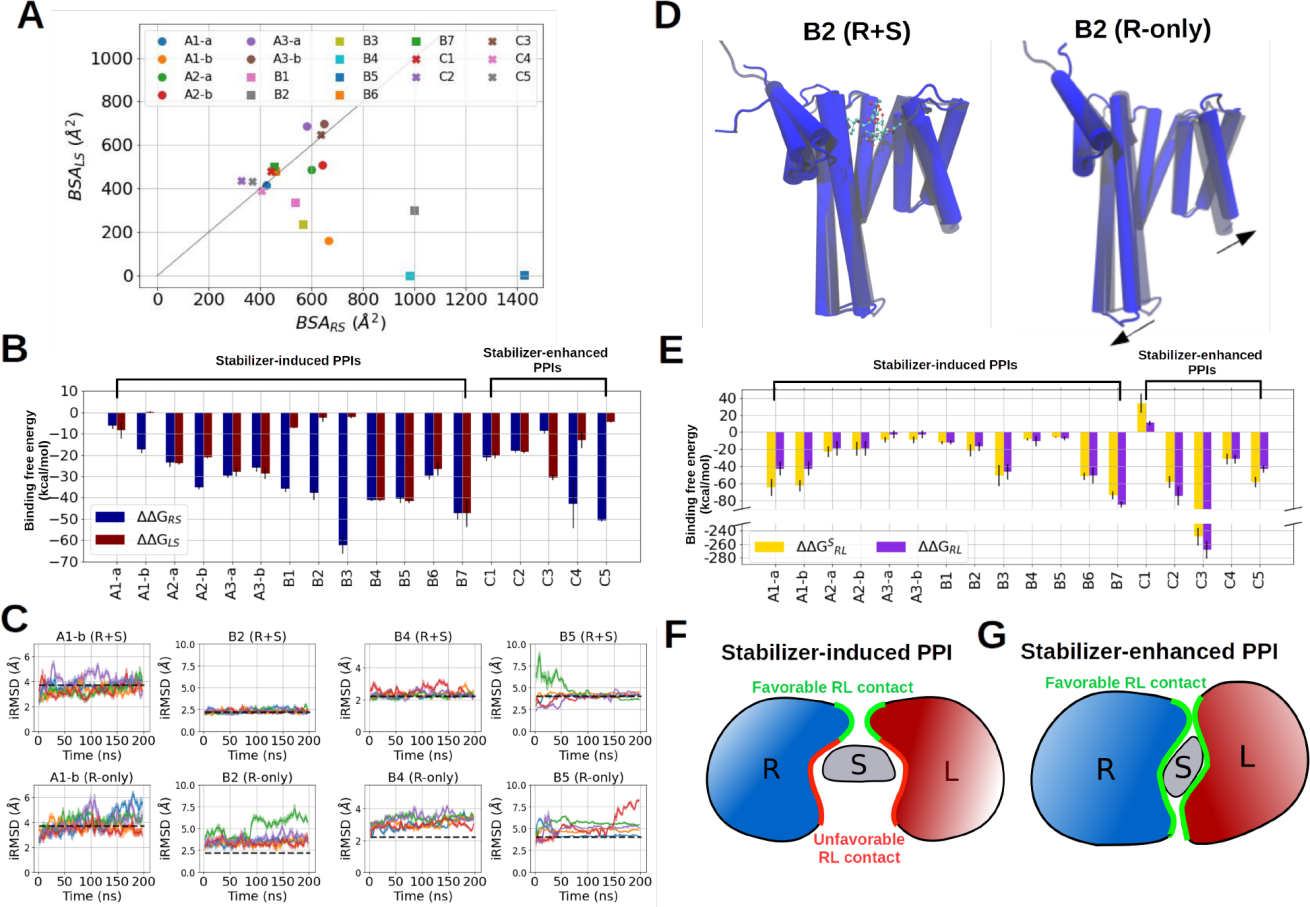
Binding free energy and interface stability calculations with MD simulations. (A) Buried surface area between the ligand and stabilizer BSA_LS_ and between the receptor and stabilizer BSA_RS_ from the RLS crystal structures listed in Table 1. (B) Binding free energy between receptor and stabilizer (dark blue) and between ligand and stabilizer (dark red). (C) Interface root-mean-square deviation (iRMSD) of the receptor in the stabilizer-bound form (top) and the receptor-only form (bottom). Five different colors indicate five independent simulations. Data points represent sliding window averages over 2 ns with the standard deviation indicated by the shaded area. (D) Schematics of the allosteric effect of PPI stabilizer exemplified with B2 receptor with the stabilizer (left) and without the stabilizer (right). The receptor of the crystal structure is shown in transparent, and the last frame of the 200 ns MD simulation is shown in solid representation. PPI stabilizer is shown in CPK representation, and the movement of the receptor–ligand binding interface is indicated by the black arrows. (E) Binding free energy between receptor and ligand in the stabilizer-bound form (yellow) and the stabilizer-free form (purple). (F) Schematic of stabilizer shielding the unfavorable RL contacts of stabilizer-induced PPIs. (G) Schematic of stabilizer shielding the favorable RL contacts of stabilizer-enhanced PPIs.

A stabilized PPI contains a receptor protein (R), a ligand protein (L, typically the smaller protein partner), and a stabilizer (S) and is abbreviated as an RLS complex. Similarly, a PPI without a stabilizer is abbreviated as an RL complex. The 15 PPIs with available three-dimensional (3D) structures are split into 3 data sets. Set A contains 3 stabilizer-induced PPIs where the stabilization effect of two stabilizers on the same R-L pair are experimentally measured and cocrystallized. These were selected to investigate how the experimentally measured stabilization efficiency is correlated with the structural, dynamical, and thermodynamic properties of the RLS complexes. Set B is formed by an additional 7 stabilizer-induced PPIs (structures known in complex with one stabilizer) and 5 stabilizer-enhanced PPIs from set C (for which 3D structures also in the absence of stabilizer are known). In example A1, the complex between protein 14-3-3 and the C-terminal domain of PMA2 can be induced by the dipeptide Epibestatin and (A1-a, *K*_RL,eff_ = 1.8 μM) and by Pyrrolidone 1 (A1-b, *K*_RL,eff_ = 80 μM).^42^ In example A2, the dimerization of the weakly dimerizing protein immunologic regulators programmed death 1 (PD-1) ligand (PD-L1, melting temperature *T*_m_ = 35.4 °C) is enhanced by small molecules BMS-202 (A2-a, *T*_m_ = 48.4 °C) and BMS-8 (A2-b, *T*_m_ = 44.8 °C).^43^ Similarly, the dimerization of bromodomain-containing protein 4 (BRD4) can be also induced by small triazolopyridazine-containing molecules compound 6 (A3-a, p*K*_RL,eff_ = 8.1) and compound 6 (A3-b, p*K*_RL,eff_ = 7.2)^44^ in example A3. Notably, although different binding poses of the stabilizers in A1 and A2 are observed, the RL complexes adopt similar conformations upon stabilizer binding, as indicated by the high TM-scores in Table 1. Despite differences in the interaction surface of the different stabilizers it is useful to first compare the sizes of the interfaces the stabilizers share with both protein partners. Hence, we first calculated the buried surface area (BSA) of RS and LS of the studied 18 RLS complexes (for a quick estimation of contacts between stabilizers and proteins, Figure 3A). For most stabilizers, a similar BSA is shared with both protein partners, indicating a similar number of contacts between the stabilizer and both proteins (Table 2). However, surprisingly, although A2-a and A3-a are more potent stabilizers than A2-b and A3-b, respectively, they form less BSA with R and L. Furthermore, we observed four stabilizers in A1-b, B2, B4, and B5 having only a little or no contact with the ligand, which violates the requirement for the dual-binding mechanism discussed above.

**Table 2:** Calculation of Mean Interaction Energies in the RL and RLS Complexes^a^

	PDBID	BSA_LS_/BSA_RS_	ΔΔ*G*_RS_ (kcal/mol)	ΔΔ*G*_LS_ (kcal/mol)	|ΔΔ*G*_RS_ – ΔΔ*G*_LS_| (kcal/mol)	ΔΔ*G*_(RL)S_ (kcal/mol)
Set A. Stabilizer-Induced PPI with 2 stabilizers						
A1	(a) *3m50*	*0.98*	***–6.27 ± 1.61***	*–8.43 ± 3.97*	*4.54 ± 3.36*	*–17.16 ± 3.52*
	(b) 3m51	0.24	–17.45 ± 1.7	**0.11 ± 0.57**	17.56 ± 2.22	–24.46 ± 2.5
A2	(a) *5j89*	*0.81*	***–23.68 ± 2.06***	–*23.79 ± 0.66*	*1.78 ± 1.07*	*–54.89 ± 2.59*
	(b) 5j8o	0.79	–35.16 ± 0.97	–**21.08 ± 0.52**	14.08 ± 0.79	–62.58 ± 1.53
A3	(a) *5ad3*	*1.18*	*–29.64 ± 0.87*	***–27.95 ± 2.01***	*2.3 ± 1.2*	*–61.94 ± 2.93*
	(b) 5ad2	1.08	**–25.93 ± 2.02**	–28.7 ± 2.65	3.77 ± 2.97	–58.47 ± 3.24
Set B. Other Stabilizer-Induced PPI						
B1	6ygj	0.63	–43.03 ± 11.45	**–12.93 ± 3.94**	30.11 ± 15.11	–87.96 ± 4.4
B2	2o98	0.3	–50.51 ± 0.78	****–**4.35 ± 0.34**	46.16 ± 0.72	–64.26 ± 2.36
B3	5fqd	0.42	–35.9 ± 1.43	**–7.33 ± 0.24**	28.57 ± 1.47	–48.65 ± 1.53
B4	4mdk	0	–37.84 ± 3.22	**–2.66 ± 1.67**	35.18 ± 3.51	–45.46 ± 3.68
B5	1kkq	0	–62.43 ± 3.84	**–2.17 ± 0.75**	60.26 ± 4.31	–66.32 ± 3.73
B6	3u15	1.04	**–41.06 ± 0.54**	–41.25 ± 0.6	0.78 ± 0.59	–93.54 ± 0.72
B7	3vbg	1.11	–**40.61 ± 1.99**	–41.84 ± 0.95	2.19 ± 1.79	–93.43 ± 2.25
Set C. Stabilizer-enhanced PPI						
C1	3tct	1.08	–29.84 ± 1.67	**–26.53 ± 3.12**	3.31 ± 2.7	–61.82 ± 2.93
C2	3ko0	1.34	**–47.25 ± 3.22**	–47.39 ± 6.29	8.81 ± 3.35	–104.13 ± 3.42
C3	3bbr	1.02	–21.14 ± 1.79	**–20.29 ± 1.41**	1.77 ± 1.57	–53.64 ± 2.43
C4	6mg5	0.96	–17.93 ± 0.86	–18.61 ± 0.61	1.25 ± 0.26	–44.36 ± 1.06
C5	4j9z	1.17	–8.79 ± 1.12	–30.69 ± 1.2	21.9 ± 2.16	–42.76 ± 0.65

a Note: Buried surface area and interaction free energy were calculated with the MMGBSA end point method. The more potent stabilizers in set A are highlighted in italic font. The calculated interaction free energies of the weaker interaction with the stabilizer are shown in bold font. The error shows the standard deviation of five values obtained from five independent simulations.

To estimate the interaction more accurately, we perform MD simulations and calculated the interaction free energies ΔΔ*G*_RS_, ΔΔ*G*_LS_, and the total interaction free energy between the protein partners and the stabilizer ΔΔ*G*_(RL)S_ (Table 2 and Figure 3A) using the Molecular Mechanics Generalized Born surface area (MMGBSA) end point method (see Methods). Indeed, a comparison between the more potent and less potent stabilizers in set A shows that the weaker stabilizer-binding partner, namely, max{ΔΔ*G*_RS_, ΔΔ*G*_LS_}, has a more favorable calculated interaction to the more potent stabilizers (highlighted in italic font in Table 2) than to the less potent stabilizers.

However, stabilizers in complexes B2, B4, and B5 do not follow this rule and also bind to the ligand-protein partner with calculated interaction free energies ΔΔ*G*_LS_ less than −5 kcal/mol (including also A1-b), which is the minimum binding free energy required to induce a PPI complex with *K*_RL,eff_ at a μM level at [*R*_0_] = [*L*_0_] = 1 μM, according to the theoretical model (Figure 1D), implicating another possible PPI-inducing mechanism (Table 2, Figure 3B).

In order to understand how these four stabilizers induce the RLS complexation without bivalent binding to both protein partners, we removed the ligand proteins and performed 200 ns MD simulations with (R + S) and without stabilizers (R-only). We found that the interface residues of the receptors, e.g., the residues in contact with the ligand protein in the RLS complexes, remain stable only in the presence of stabilizers but become much more mobile when the stabilizers are removed, indicated by increased interface root-mean-square deviation (iRMSD, Figure 3C–D). Hence, in these cases, the stabilizers bind to one protein partner (receptor) and stabilize the bound conformation, and in turn, allosterically stabilize PP complex formation.

We next calculated the direct interaction between receptor and ligand with the stabilizer  and without (ΔΔ*G*_RL_) the stabilizer (see Methods). Figure 3E shows that the calculated RL interaction is weakened in most stabilizer-induced PPIs when the stabilizers are removed but is stronger or similar in most stabilizer-enhanced PPIs without stabilizers. In order to understand this effect, we performed residue-wise energy decomposition analysis on the RL complexes simulations and identified the favorable RL contacts (indicated by negative binding energies) and unfavorable RL contacts (indicated by positive binding energies, SI Appendix, Figures S1 and S2). Interestingly, the unfavorable RL contacts in the stabilizer-induced PPI complexes are often formed by the residues responsible for stabilizer binding (SI Appendix, Figures S1 and S2).

Thus, in addition to the dual-binding mechanism, stabilizers can also contribute to more favorable binding by shielding the unfavorable RL interactions. This may explain why the conformations of receptor and ligand in set A tend to form a similar conformation upon stabilizer binding where favorable RL contacts and druggable pockets near the unfavorable RL contacts can be formed simultaneously (Table 1). When stabilizers are removed from these RLS complexes, these unfavorable contacts are exposed to their binding partner, causing interface distortions. Such an effect was observed in complexes A3, B6, and B7 within 50 ns simulations (Figure 3F, SI Appendix, Figure S3, and SI Appendix, Figure S4). In contrast, the interfaces of the stabilizer-enhanced PPIs remained stable without stabilizers (Figure S5). The binding of these stabilizers involves many favorable RL contacts (Figure 3G and SI Appendix, Figure S6) and is therefore more likely to disturb the direct RL interactions.

### MD Simulations Reveal Hidden Stabilizer-Binding Pockets

**Figure 4:**
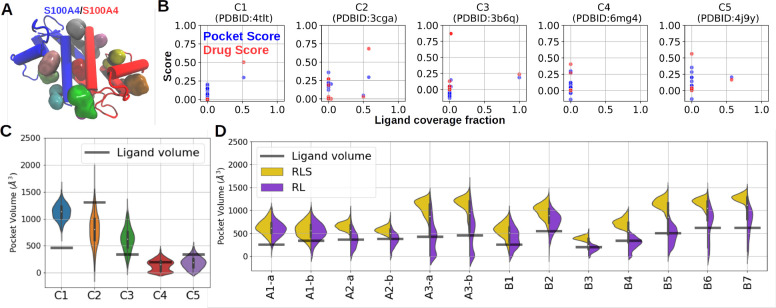
Interface pocket detection and discovery with MD simulations. (A) A scheme of the pockets revealed by Fpocket from the C2 RL complex (PDBID: 3cga). (B) Correlation of the ligand coverage fraction and the scores provided by Fpocket. (C) Distribution of the volume of the stabilizer-binding pocket in set C in MD simulations. (D) Distribution of the volume of the stabilizer-binding pocket in sets A and B in MD simulations in the stabilizer-bound state (RLS complex, yellow) and the stabilizer-free state (RL complex, purple). The volume of ligands is calculated from http://www.scfbio-iitd.res.in/Sanjeevini/Molecular-volume-calculator.php and indicated with transparent lines.

In order to discover potential PPI stabilizers in the scheme of structure-based drug design (SBDD), it is crucial to identify useful potential ligand-binding pockets. Structural comparison has shown that PPI stabilizers can bind into an interface pocket of an RL complex without deforming the conformation (Table 1), offering an opportunity for virtual screening. We used Fpocket4.0^55^ to probe druggable pockets of the natural RL complexes and detected at least 10 pockets in each RL complex (Figure 4A). Although the dual-binding mechanism already excludes the noninterface pockets, performing virtual screening through all possible interface pockets is still computationally expensive, and therefore, a reliable approach to rank the interface pockets is desired to narrow down the sampled phase space. To examine whether the scores provided by Fpocket, e.g., the drug score and pocket score, can accurately distinguish the stabilizer-binding pocket from other interface pockets, we calculated the ligand coverage fraction of each pocket (see Methods). Besides the C4 complex, all stabilizer-binding pockets are fully or partially detectable from the RL complex and well ranked by both pocket score and drug score, indicating that it is a reliable approach to identify potential stabilizer-binding pockets solely on the basis of the RL structure (Figure 4B). Further the use of MDpocket to track the dynamics of stabilizer-binding pockets from the simulation (see Methods) reveals that all pockets exhibit a breathing motion with a considerable range of volume sampled in the simulations (Figure 4C and time series shown in SI Appendix, Figure S7). Importantly, the stabilizer-binding pocket hidden in the RL crystal structure of the C4 complex occasionally expands to a volume large enough to accommodate the stabilizer with a rapid fluctuation in pocket volume (Figure 4C and SI Appendix, Figure S7). Upon removal of the stabilizer from the RLS complexes, the volume of the stabilizer-binding pocket showed in a few cases little changes during simulations. However, in many cases, a reduced pocket size distribution sampling frequently pocket volumes smaller than the ligand volume was observed (Figure 4D). In the A3, B6, and B7 cases, the removal of the stabilizer resulted in a distortion of the RL interface such that the stabilizer binding pocket became completely undetectable (SI Appendix, Figures S3, S4, S8, and S9). Note, interface distortion and RL complex dissociation are expected in all stabilizer-induced PPI if simulations were extended to much longer (e.g., microsecond) time scales due to the very weak interaction in these cases.

### Application of the Dual-Binding Mechanism to the *In Silico* Identification of PPI Stabilizers

Our systematic study of 18 PPI stabilizers reveals PPI stabilization mechanisms and the dynamics of the stabilizer-binding pockets. In particular, the dual-binding mechanism derived from the starting theoretical model and supported by experimental results shows that the binding affinity between the stabilizer and the weakly interacting protein partner plays a decisive role in the RLS complexation process. Hence, it may provide a better ranking than the conventional structure-based drug design (SBDD) workflow, which usually only considers the total calculated interaction energy between the compound and the target of interest.

**Figure 5:**
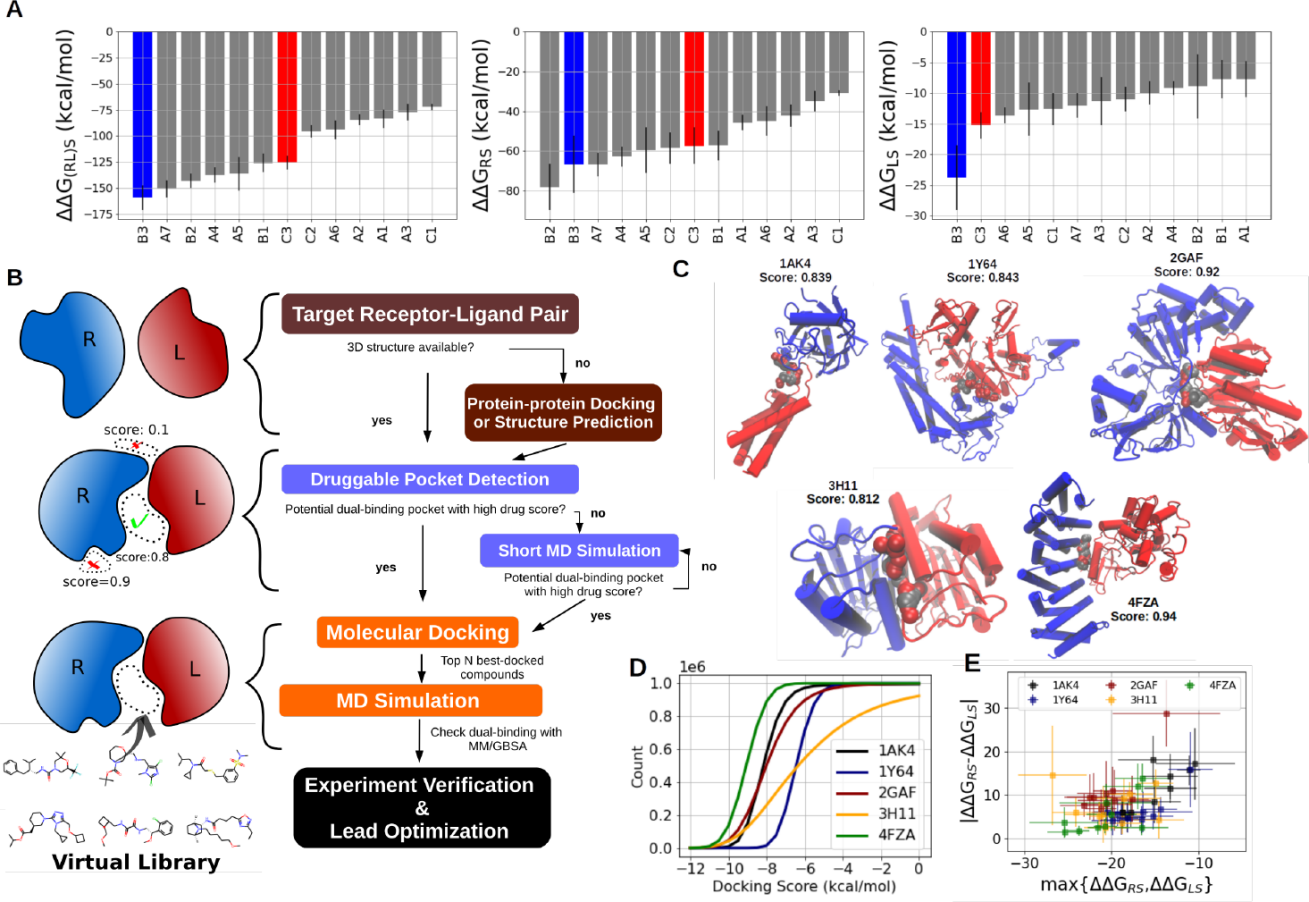
Application of the dual-binding mechanism to PPI stabilizer discovery. (A) Reranking of 13 potential 13-4-4/ChREBP stabilizers using the total binding energy (left, Δ*G*_(RL)S_), binding energy to the receptor (middle, Δ*G*_RS_), and binding energy to the ligand (middle, Δ*G*_LS_). Compounds B3 and C3 are the most potent stabilizers with EC_50_ values of 0.7 μM and 45 μM,^28^ respectively. (B) *In silico* stabilizer discovery workflow. Brown boxes show the PPI structure collection. The blue boxes indicate the pocket detection phase, and the orange boxes point to *in silico* interaction free energy evaluation with molecular docking and MD simulations. (C) Structures of 5 PPI complexes with potential stabilizer pockets (receptor proteins in blue and ligand proteins in red cartoon). The pocket is defined by the van der Waals sphere at the interface with the red and gray spheres representing the polar and apolar pocket probes. (D) The population of the docking score of the 1 million compounds screened through the 5 example PPI complexes. (E) Binding free energy difference |ΔΔ*G*_LS_ – ΔΔ*G*_RS_| vs the weaker stabilizer-binding energy maxΔΔ*G*_RS_,ΔΔ*G*_LS_} of the ten ligands with the best docking score at each PP interface. The numerical values of ΔΔ*G*_RS_, ΔΔ*G*_LS_, |ΔΔ*G*_LS_ – ΔΔ*G*_RS_|, and ΔΔ*G*_(RL)S_ are listed in SI Appendix, Tables S1–S5.

We tested the dual-binding ranking approach on the 14-3-3/ChREBP complex with 13 compounds preselected by Sijbesma et al.^28^ through a virtual screening approach (SI Appendix, Figure S10A). We performed molecular docking, MD simulations, and MM/GBSA calculations to check the dual-binding mechanism of these compounds (see Methods, Supplementary Figure 10B). For all compounds, a stronger calculated interaction with the receptor compared with the ligand was observed (Figure 5A). According to the proposed dual-binding mechanism, the interaction of the compound and the weaker ligand partner then plays a more decisive role in stabilization efficiency. Indeed, an improvement in ranking performance (compared to the experiment) was found when the compounds are ranked using the binding affinity to the ligand instead of the receptor or vs total binding energy (Figure 5A).

To this end, we propose an *in silico* protocol adapted for PPI stabilizer discovery (Figure 5B). A 3D structure of the desired RL pair needs to be available, e.g., experimental structure or based on protein–protein complex modeling. Next, Fpocket can be used to probe and rank druggable pockets at the PPI interface. One can further assess the stability of the pocket or explore hidden pockets by performing MD simulations. Once a suitable binding pocket is identified, virtual screening can be conducted with molecular docking programs to screen through a large chemical database (e.g., following the protocol described in the Methods). Compounds with high docking scores can then be submitted to MD simulations to study the binding stability and interaction free energy. At this step, it is important to check if the compound-protein interaction is of a similar magnitude with both protein partners (desired to optimize the stabilizing effect according to the dual binding mechanism). Finally, compounds that fit the criteria can be submitted to experimental validation and lead optimization.

As a demonstration, we implemented the protocol to a subset of protein docking benchmark set 5.0,^56^ that contains 226 high-quality protein complexes. Pocket detection revealed that among 226 tested complexes, 223 complexes (98.7%) have at least one pocket consisting of 5 atoms from each side of the interface, 173 complexes (76.5%) have at least 10 atoms from each side of the interface, and 57 complexes (25.2%) have at least 20 atoms from each side of the interface (SI Appendix, Figure S11A–B). We further extracted the best-scored interface pockets composed of at least 5 atoms from each protein, and several pockets were identified, some with pocket scores and drug scores higher than the known stabilizer-binding pockets discussed in the previous sections (Figure 4B, SI Appendix, Figure S11C–D).

Among these pockets, we chose 5 interface pockets with high drug scores, including PDBID 1AK4, 1Y64, 2GAF, 3H11, and 4FZA, for virtual screening (Figure 5C). We performed molecular docking on these five interface pockets with 1 million compounds selected from the ZINC20 database^57^ (see Methods). Besides the pocket predicted with the poorest drug score (score = 0.812, PDBID 3H11), all pockets can accommodate over 75% of the screening compounds with a docking score better than −6 kcal/mol, indicating good druggability preselected with drug scores (Figure 5D). The top-ranked 10 docked compounds of each protein complex and their docking score are listed in SI Appendix, Figures S12–S16 and were submitted to MD simulations for interaction free energy calculation (SI Appendix, Tables S1–S5). As demonstrated in the present study, an ideal PPI stabilizer usually possesses a strong binding affinity to the weaker stabilizer-binding partner, indicated by a low max{ΔΔ*G*_RS_, ΔΔ*G*_LS_}, and a similar binding affinity to both proteins, indicated by a low |ΔΔ*G*_RS_ – ΔΔ*G*_LS_|. Among the 50 selected compounds, 34 compounds (68%) possess a max{ΔΔ*G*_RS_, ΔΔ*G*_LS_} lower than −15 kcal/mol, and a |ΔΔ*G*_RS_ – ΔΔ*G*_LS_| smaller than 10 kcal/mol (Figure 5E). In particular, the best three compounds at the PP interface of 4FZA (compound 5, compound 8, and compound 9, SI Appendix, Table S5) all exhibited a max{ΔΔ*G*_RS_, ΔΔ*G*_LS_} lower than −24 kcal/mol, and |ΔΔ*G*_RS_ – ΔΔ*G*_LS_| smaller than 4 kcal/mol, and are expected to be promising stabilizers. Together with the dual-binding mechanism of stabilizers, our results provide not only a prediction of potential PPI stabilizers but also a guide for further lead optimization, for example, by improving the RS interaction of compound 3 in PDBID 1AK4 or by improving the LS interaction of compound 4 in PDBID 3H11 (SI Appendix, Table S1 and Table S4).

## Discussion

To facilitate the development of the PPI stabilizer discovery, it is essential to understand how these compounds stabilize PP complexes. In the present study, we have case-studied the binding behavior of 18 stabilizers on their corresponding PP complexes using MD simulations, interaction free energy calculations, and pocket detection techniques. Stabilizers can facilitate PPI complex formation through the dual-binding mechanism and/or conformational stabilization of the bound receptor (or ligand) structure.^9−11^ The more common optimal dual-binding mechanism requires the stabilizer to interact approximately equally with the protein partners. The effective stabilization depends predominantly on the partner that binds more weakly to the stabilizer. Furthermore, stabilizers can shield the unfavorable contacts between a noninteracting RL pair while not affecting the favorable contacts. We also found that a fraction of stabilizers that do not fulfill the dual-binding mechanism may also support PPI complexation by stabilizing the interface of one protein to a conformation ready for partner protein binding. The latter more complex allosteric mechanism could, in principle, be detected by extensive MD simulations of a protein partner in the presence and absence of the stabilizer. However, the result of such simulations can be sensitive to force field artifacts, and due to the computational demand, it may not be useful for investigating many compounds. Hence, in practical design efforts, it might be most promising to focus on identifying putative stabilizer compounds that follow a dual-binding mechanism.

Indeed, our analysis of the stabilizer-binding pockets in experimental structures without stabilizers demonstrated that most stabilizer-binding pockets can be readily detected. Those pockets hidden in the RL complexes can show up in short MD simulations. Furthermore, interface pockets that can accommodate ligands, allowing stabilization by the dual-binding mechanism, could be detected in the majority of cases in a large set of known PP complex structures. Based on our analysis, we demonstrated that the dual-binding mechanism can be useful to identify potent stabilizers and propose a protocol for PPI stabilizer discovery. We applied the protocol to 5 test cases and can suggest potential PPI stabilizers for five protein–protein complexes. Combining the pocket-scoring technique and dual-binding criteria, our SBDD workflow can efficiently identify druggable interface pockets and estimate the stabilizing efficiency *in silico*.

Ideally, with advances in structural prediction and protein–protein docking techniques,^30−35^ one can design molecules that induce the complexation of selected proteins. For a given interaction geometry, our protocol could also be useful for the identification of such compounds that induce a PPI or support a very weak association. Our work is the first computational study systematically characterizing the binding behavior across different kinds of PPI stabilizers. We anticipate that our study will facilitate future developments for the discovery of PPI stabilizers.

## Methods

In this study, in total, 23 protein complex structures were used from the RCSB PDB database, including 18 RLS complexes and 5 RL complexes (Table 1). Due to missing side chains and short backbone segments in some proteins (and some mutated residues), the comparative modeling software MODELER^58^ was used with the wild-type sequences listed in Table 1. This includes the restoration of the mutations N755S in PDBID 3bbr, T686A in PDBID 3b6q, L22Q, T51D, V52I in the ligand of PDBID 3o98, S13A, T30D, V31I in the ligand of PDBID 3m50 and 3m51, C6S in PDBID 3u15, and L9E and 3vbg. The RL complexes of sets A and B are generated by stripping the stabilizers from the corresponding RLS complex. Because of the high structural similarity, A1-a, A2-a, and A3-a were taken to represent the RL complexes of A1, A2, and A3, respectively. Besides the 14-3-3 protein and its ligands, where the terminal residues are as well interacting with each other and the stabilizers, the N-terminus and C-terminus are capped with the neutral capping group ACE and NME with Ambertools 20,^59^ respectively. To maintain the conformations of the complex, two calcium ions are kept in the S100A4 homodimer and CaM/CaMBD2a complex, four glutamic acids are kept in the iGluR2 homodimer, and a zinc ion is kept in the CK1α/CRL4. Also, disulfide bonds in PD1L/PD1L and lambda-6A/lambda-6A complexes are preserved.

Each system was solvated in a box 12.5 Å extended from the nearest atom of the solute with OPC 4-point water molecules and 0.15 M sodium chloride. The protonation state of the stabilizers is determined with Open Babel^60^ at pH = 7.4. The atomic interactions are described by the ff19SB force field^61^ for protein, OPC force field^62^ for water, the TIP4PEW^63^ force field for ions, and GAFF2^64^ for the stabilizers. The same procedure was also implemented to prepare the MD simulation of the 5 example complexes from protein docking benchmark data set 5.0. Five replicas of 50 ns trajectories of each RL and RLS complex and 5 replicas of 200 ns long trajectories of each RS complex and receptor-only system of PDBID 3m51, 2o98, 4mdk, and 1kkq are generated with MD simulation with the CUDA-accelerated version of PMEMD from AMBER20 package with randomly assigned initial velocities. Each simulation box underwent the minimization process of maximal 35,000 steps and was equilibrated for 75 ps under constant volume and 225 ps under constant pressure. Gradually decreasing positional restraints on protein atoms are applied during the minimization and equilibrium processes. The temperature was kept at 310 K using a Langevin thermostat, and the pressure was kept at 1 bar with a Berendsen barostat. The nonbonded cutoff distance was set to 9 Å, and the simulation was integrated with a time step of 2 fs with the SHAKE algorithm. Interface residues are defined by the residues within 5 Å of the partner protein calculated using VMD-PYTHON. RMSD and iRMSD are calculated with the starting structure as the reference. RMSF, RMSD, and iRMSD are calculated using CPPTRAJ. The last 30 ns, in a total of 75 frames, of each 50 ns simulation in the RL and RLS simulation are taken to calculate the interaction free energies between receptor, ligand, and stabilizer using molecular mechanics coupled with the generalized-Born surface area (MM/GBSA) method with an internal dielectric constant of 1 and external dielectric constant of 80. The effective Born radii are calculated with the GBOBCII model (igb = 5) in ref (65). Pocket detection was performed with Fpocket4.0 on the crystal structure of the RL complexes in set C. Each pocket consists of polar and apolar probes, as shown in Figure 5C in red and gray spheres, respectively. An atom and a pocket probe are considered in contact if the distance between the atom and the pocket probe is smaller than the sum of their radii with the radii of atoms set to H: 1.2 Å, C: 1.7 Å, N: 1.55 Å, O: 1.52 Å, F: 1.47 Å, B: 1.92 Å, P: 1.8 Å, S: 1.8 Å, Cl: 0.2 Å. L. The ligand coverage fraction is calculated as , where *N*_ligand_ ∩ *N*_pocket_ is the number of ligand atoms contacting the pocket probes, as illustrated in Figure S17. The dynamics of the stabilizer binding pocket is analyzed using MDtraj by first creating probes within 2 Å from the known stabilizers, as shown in the SI Appendix, Figure S18.

Re-evaluation of the 13 compounds performed by Sijbesma et al.^28^ was generated with Open Babel^60^ and docked with Autodock vina.^66^ The docking procedure was performed on the 3D structure from PDB 5F74. The preparation of 5*50 ns trajectories and the binding energy calculation were performed with the same procedure as mentioned above.

Considering many stabilizers in the present study possess molecular weights between 375 and 425 Da, and LogP between 2 and 4.5 (SI Appendix, Figure S19), we fetched 122,847,475 compounds fulfilling such molecular properties from the ZINC20 database^57^ with the standard highest reactivity. We further eliminated compounds with complex structures, which might cause difficulties for force field parametrization, by filtering the compounds with BertzCT complexity^67^ > 700 using RDkit,^68^ leading to 15,072.167, from which we randomly selected 1 million compounds to perform molecular docking with AutoDock-GPU.^69^

## Supporting Information Available

Data on the 18 case-study complexes (Figures S1–S9), chemical structures and the docking poses of the 14-3-3/ChREBP stabilizers (Figure S10), data used and obtained in the virtual screening protocol (Figures S11–S16 and Figure S19), and the schematics of the pocket detection technique (Figures S17–S18). The binding energies of the 10 best-docked compounds at the tested PP interfaces are listed in Tables S1–S5 (PDF)
Transparent Peer Review report available (PDF)
