# Supplementary material for: What Makes a Good Protein–Protein Interaction Stabilizer: Analysis and Application of the Dual-Binding Mechanism
Source: ACS Cent Sci. 2023 Apr 14;9(5):969–79. doi: 10.1021/acscentsci.3c00003 (PMC10214505; doi:10.1021/acscentsci.3c00003)
Supplement: Supplementary file 1 — oc3c00003_si_001.pdf [file oc3c00003_si_001.pdf]

Supporting Information: What makes a good protein-protein interaction stabilizer: Analysis and Application of the Dual-Binding Mechanism

Shu-Yu Chen and Martin Zacharias*

Center for Functional Protein Assemblies, Garching, 85748, Germany

E-mail: zacharias@tum.de

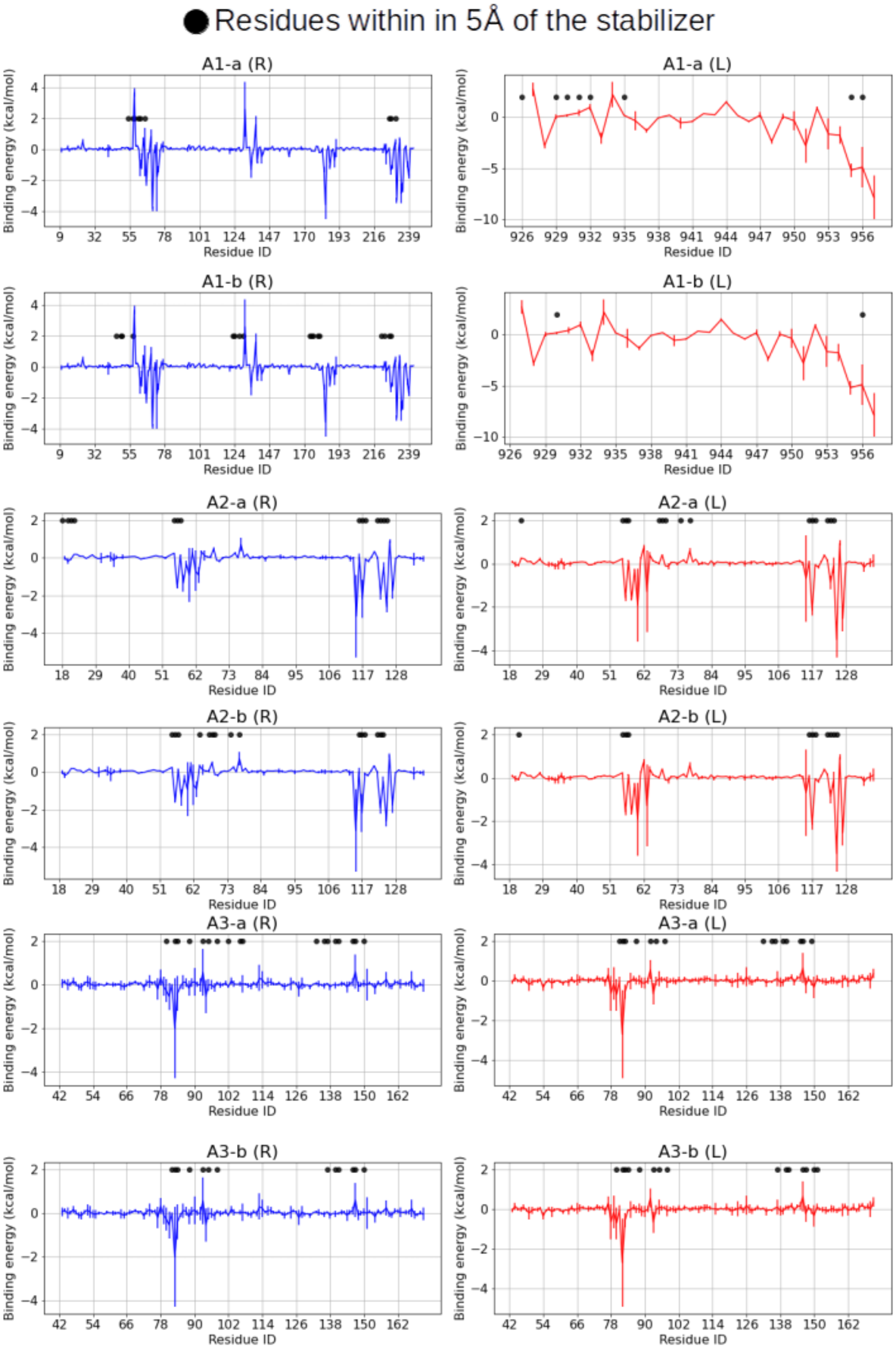

Figure S1: Residue-wise RL binding energy decomposition in set A complexes. The black dots represent the residues within 5Å of the stabilizer.

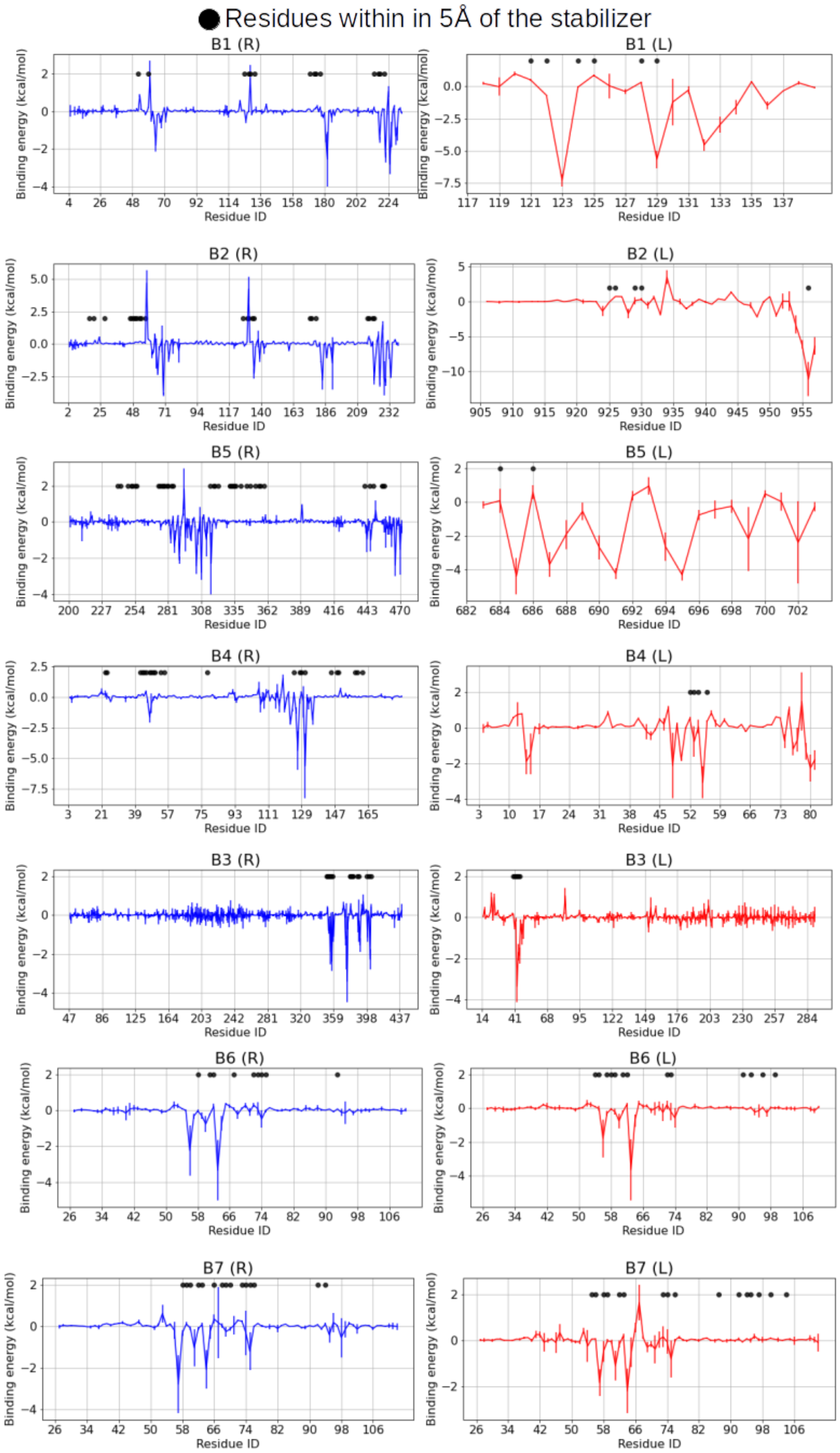

Figure S2: Residue-wise RL binding energy decomposition in set B complexes. The black dots represent the residues within 5Å of the stabilizer.

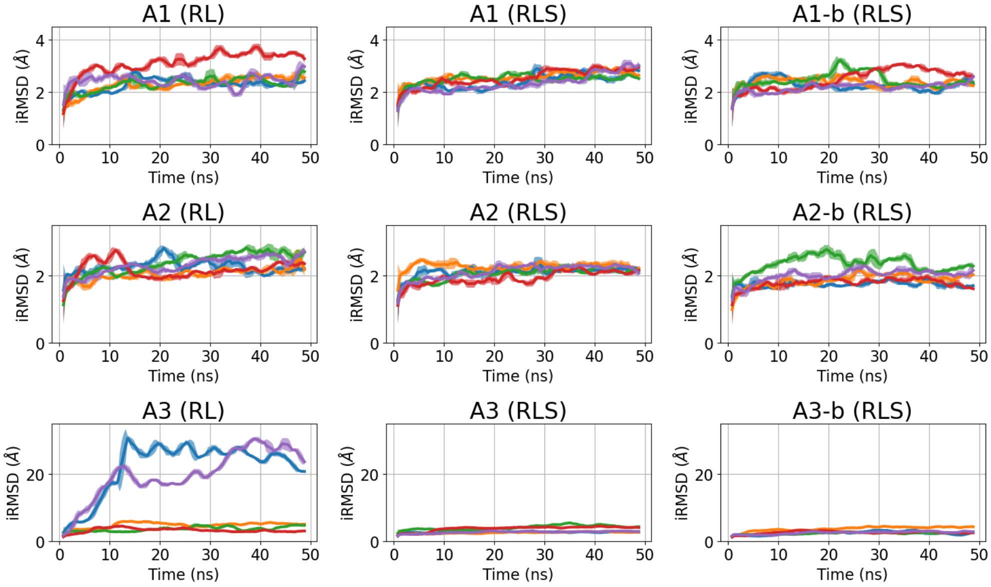

Figure S3: Interface RMSD (iRMSD) of the complexes in set A. Simulations without the stabilizers are shown in the left panel and simulations with the stabilizers are shown in the right panels. Five different colors represent five individual simulations. Data are shown as sliding averages over every 1 ns with standard deviation indicated by the shaded area.

Table S1: Interaction free energy of the 10 ligands binding to the interface of PDBID 1AK4. Structures of the ligands are shown in Figure S12. The binding affinity between the compound and the more weakly bound protein is emphasized with bold font. The 3 compounds with the lowest max$\{\Delta\Delta G_{RS},\Delta\Delta G_{LS}\}$ are expected to be the most promising stabilizers and are highlighted in gray.

Ligand	$\Delta\Delta G_{RS}$ (kcal/mol)	$\Delta\Delta G_{LS}$ (kcal/mol)	$\lvert\Delta\Delta G_{RS}$ - $\Delta\Delta G_{LS}\rvert$(kcal/mol)	$\Delta\Delta G_{(RL)S}$ (kcal/mol)
1	**-11.11 ± 1.34**	-26.75 ± 2.2	15.65 ± 3.42	-39.19 ± 0.73
2	-24.4 ± 1.69	**-19.03 ± 2.95**	5.9 ± 3.57	-50.71 ± 3.13
3	**-15.2 ± 4.04**	-33.21 ± 1.68	18.01 ± 5.5	-58.41 ± 4.53
4	**-17.77 ± 1.92**	-23.67 ± 2.85	5.89 ± 3.27	-43.5 ± 3.52
5	**-13.25 ± 3.04**	-24.68 ± 1.93	11.44 ± 3.36	-41.48 ± 5.14
6	**-15.78 ± 3.91**	-22.8 ± 3.17	8.39 ± 4.96	-45.41 ± 4.35
7	**-18.77 ± 1.17**	-24.76 ± 4.28	6.0 ± 3.87	-43.9 ± 7.04
8	-27.49 ± 2.97	**-13.28 ± 3.22**	14.22 ± 5.19	-45.91 ± 4.11
9	**-20.41 ± 2.88**	-20.8 ± 3.07	4.8 ± 3.38	-45.99 ± 2.46
10	**-11.48 ± 6.29**	-26.48 ± 5.41	17.13 ± 8.21	-36.31 ± 2.42

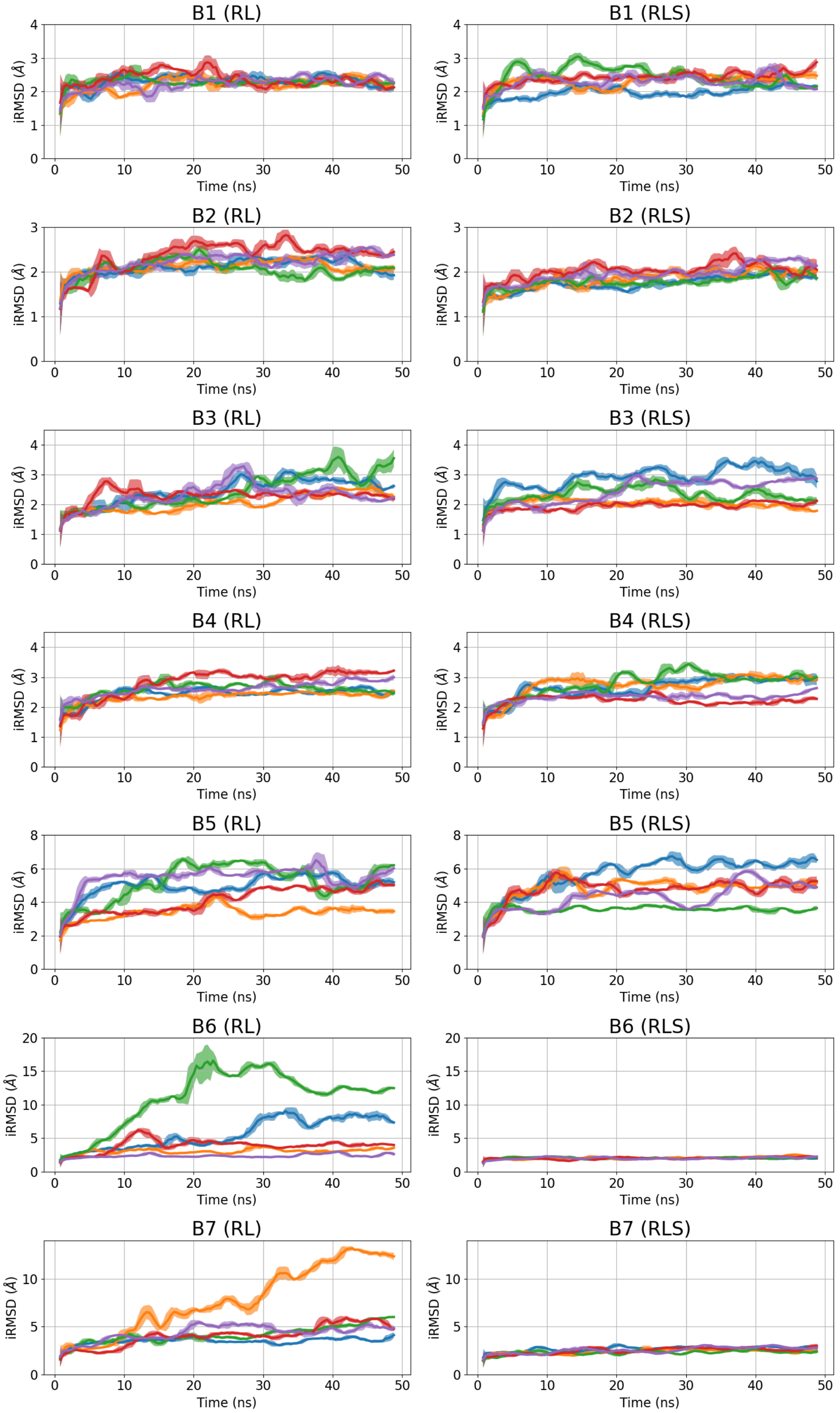

Figure S4: Interface RMSD (iRMSD) of the complexes in set B. Simulations without the stabilizers are shown in the left panel and simulations with the stabilizers are shown in the right panel. Five different colors represent five individual simulations. Data are shown as sliding averages over every 1 ns with standard deviation indicated by the shaded area.

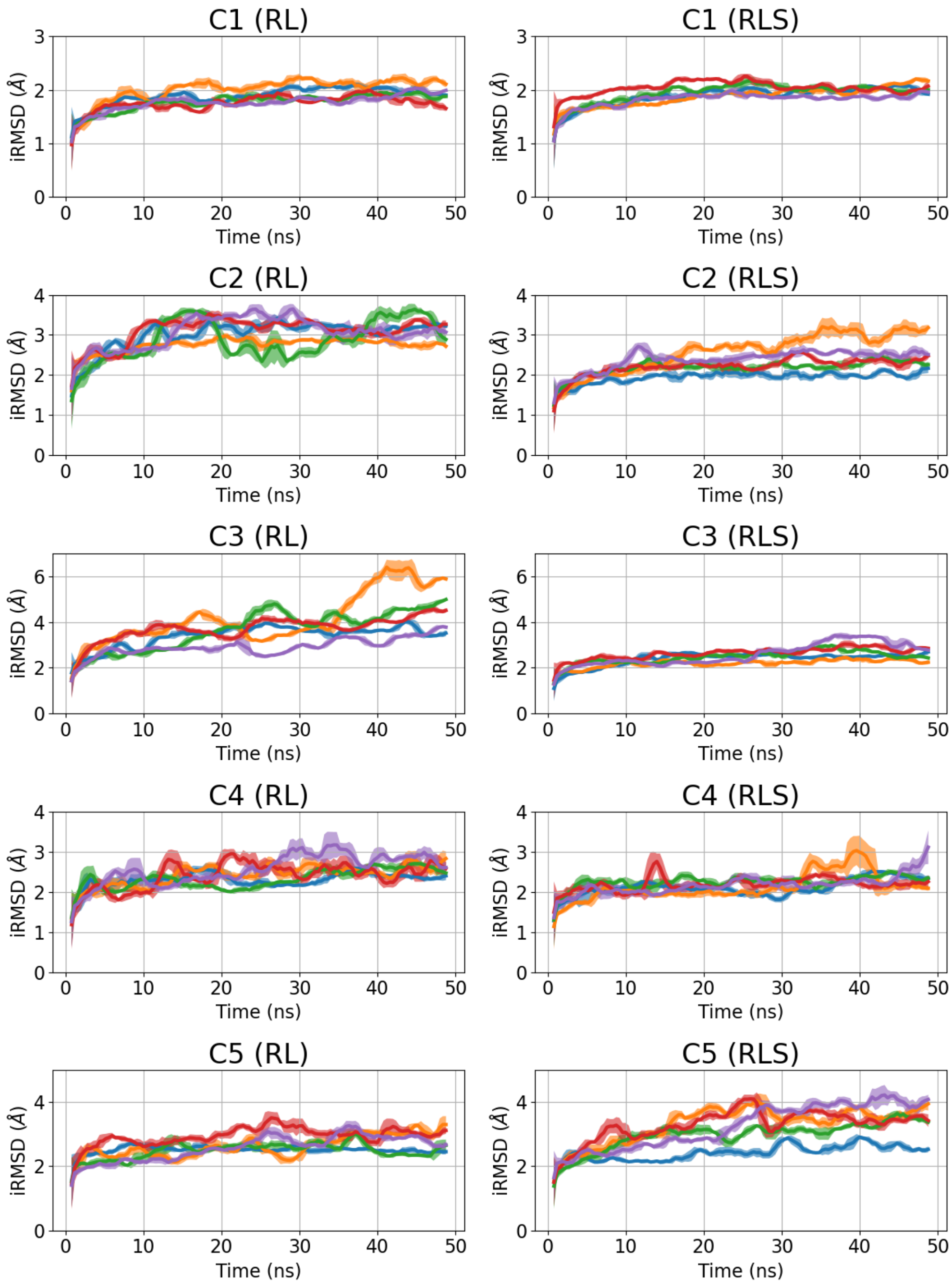

Figure S5: Interface RMSD (iRMSD) of the complexes in set C. Simulations without the stabilizers are shown in the left panel and simulations with the stabilizers are shown in the right panel. Five different colors represent five individual simulations. Data are shown as sliding averages over every 1 ns with standard deviation indicated by the shaded area.

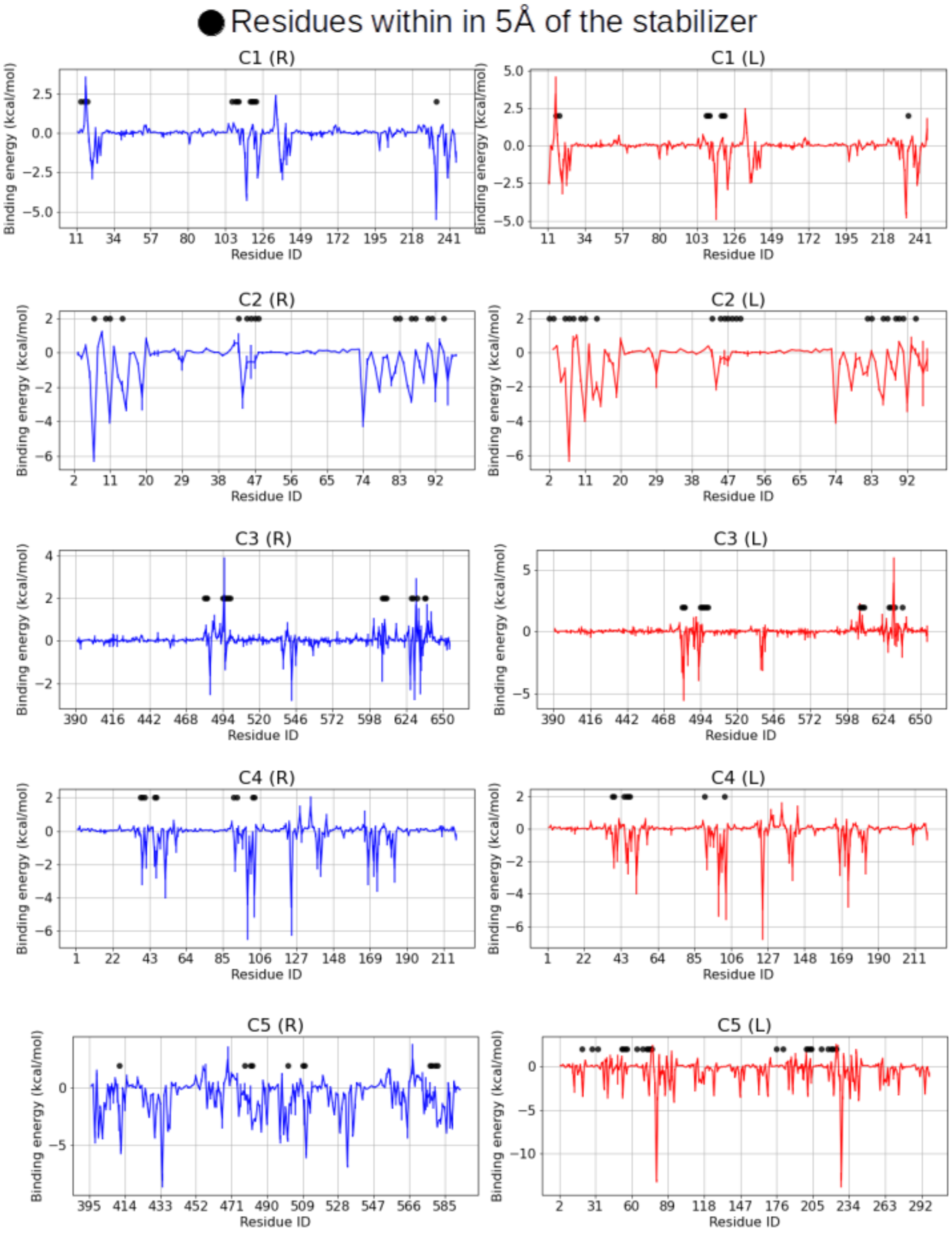

Figure S6: Residue-wise RL binding energy decomposition in set C complexes. The black dots represent the residues within 5Å of the stabilizer.

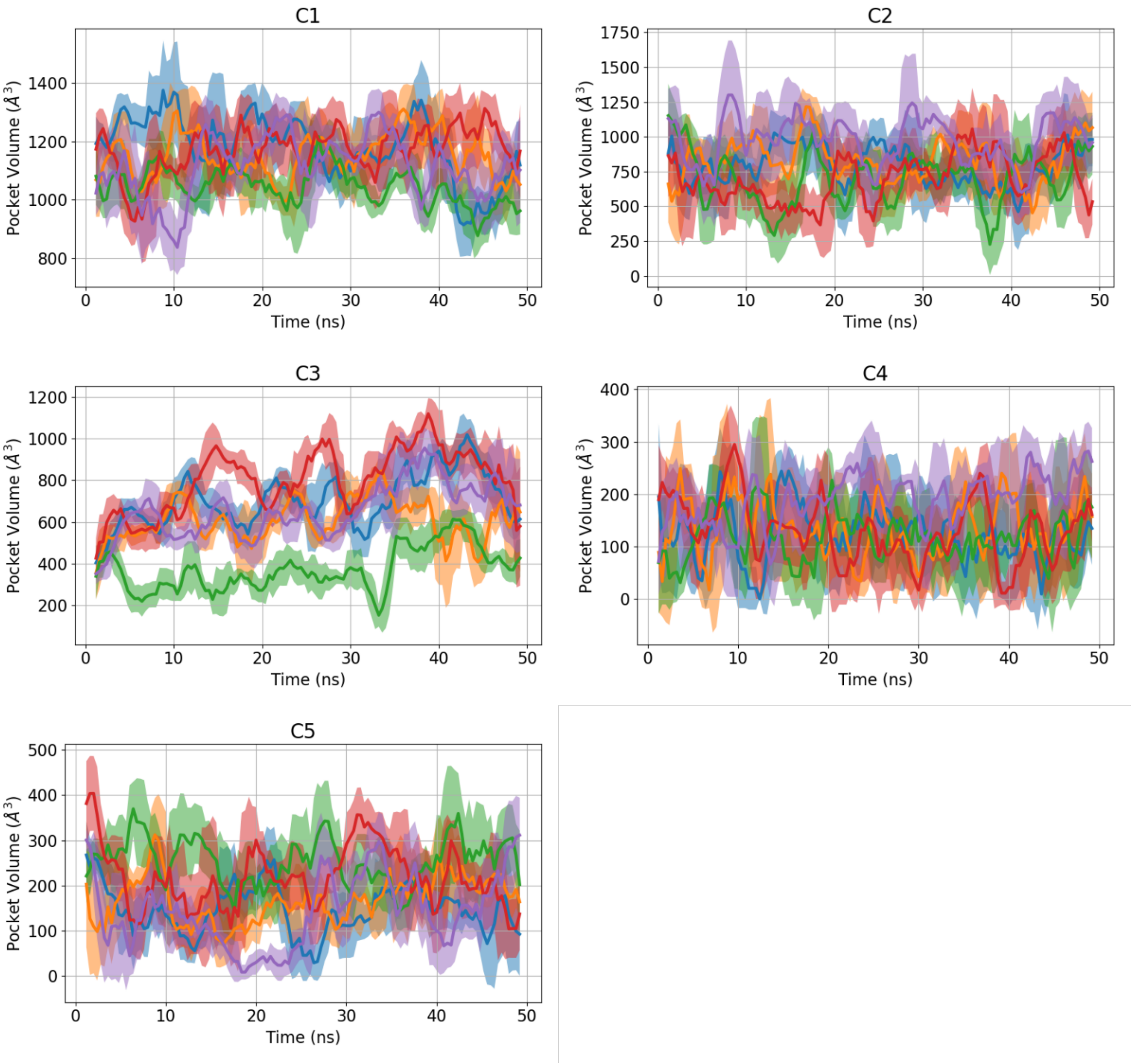

Figure S7: Time-evolution of the stabilizer-binding pocket volume in set C complexes without stabilizers. Data from five individual simulations are color-coded the same way as in Supporting Figure S5. Data are shown as sliding averages over every 1 ns with standard deviation indicated by the shaded area.

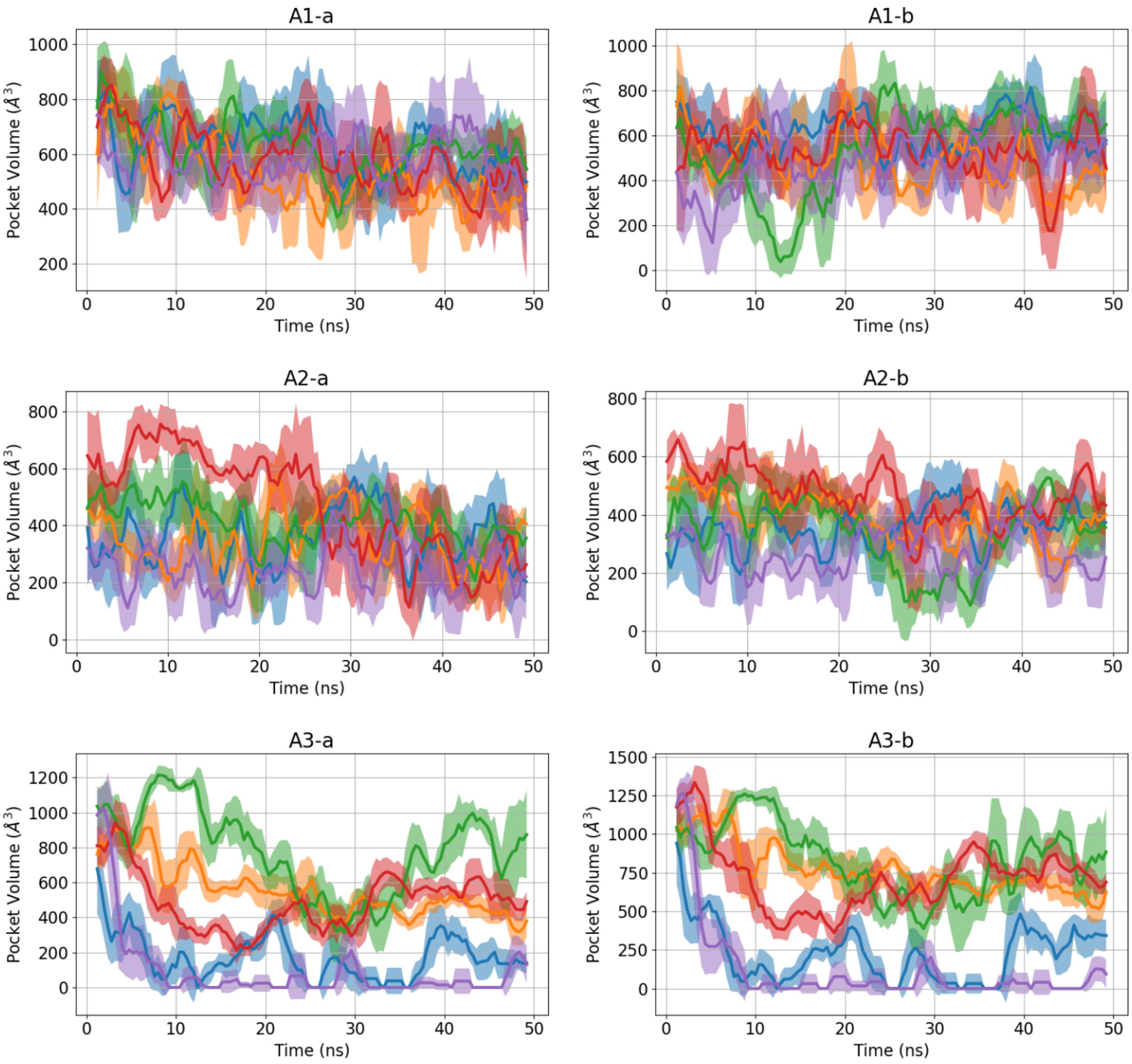

Figure S8: Time-evolution of the stabilizer-binding pocket volume in set A complexes without stabilizers. Data from five individual simulations are color-coded the same way as in Supporting Figure S3. Data are shown as sliding averages over every 1 ns with standard deviation indicated by the shaded area.

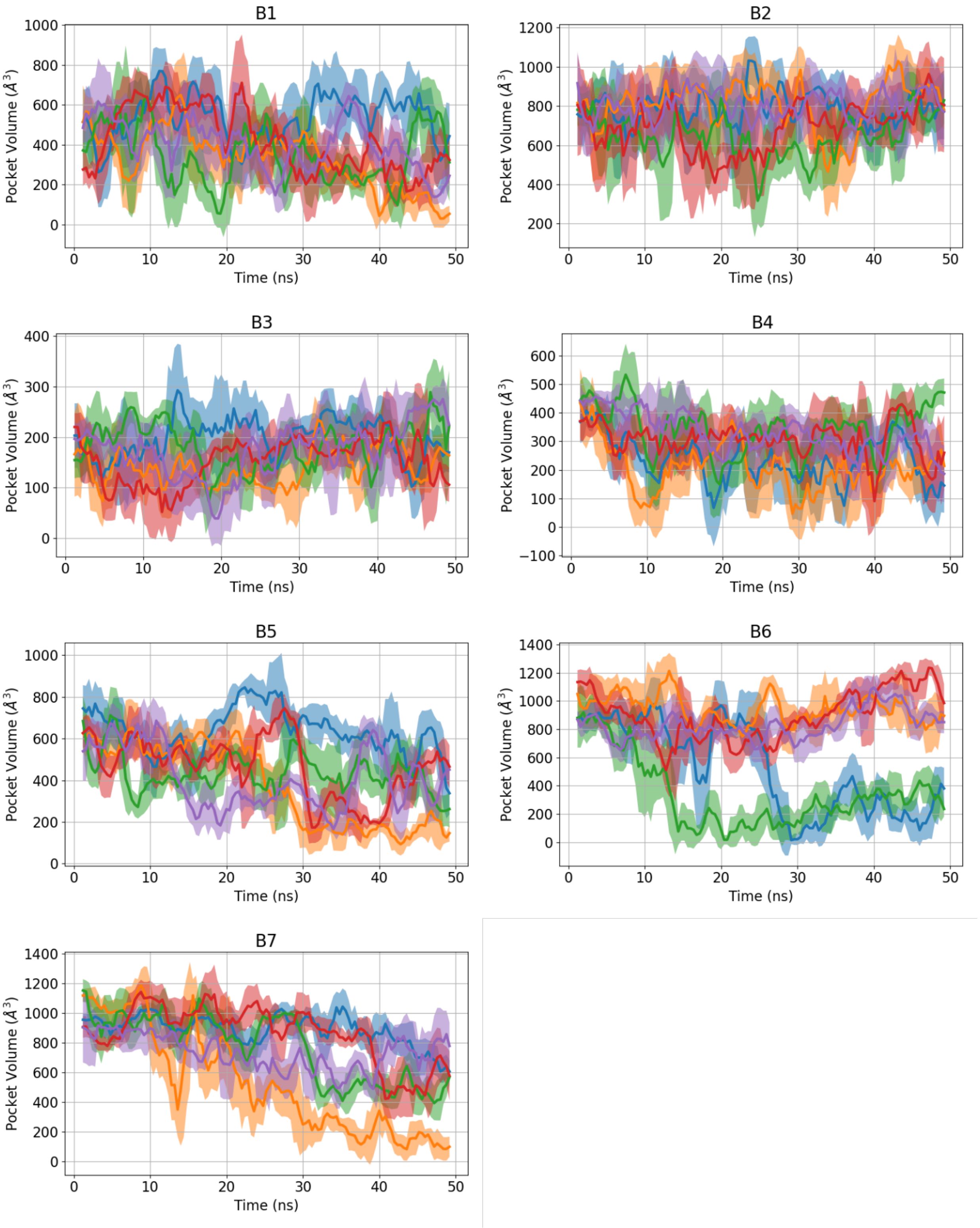

Figure S9: Time-evolution of the stabilizer-binding pocket volume in set B complexes without stabilizers. Data from five individual simulations are color-coded the same way as in Supporting Figure S4. Data are shown as sliding averages over every 1 ns with standard deviation indicated by the shaded area.

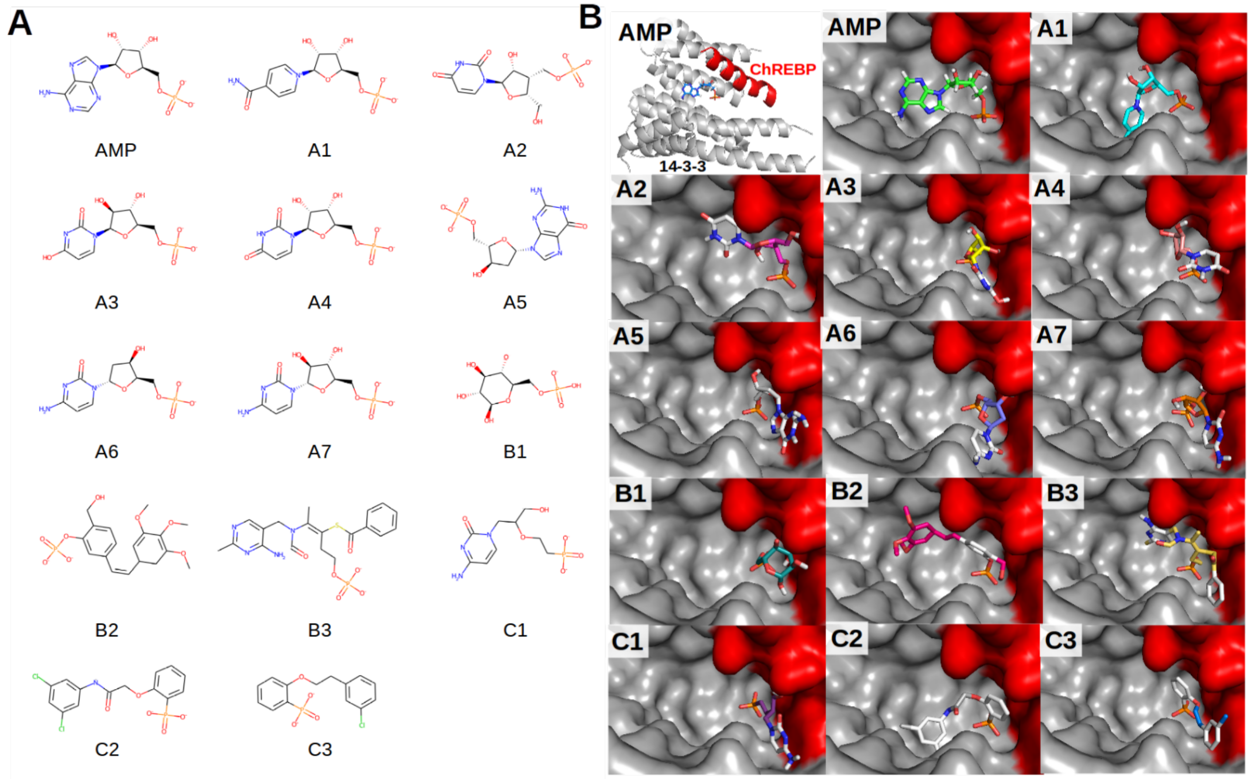

Figure S10: MD simulations with preselected compounds binding to 14-3-3/ChREBP complex. (A) Chemical structures of the 13 compounds investigated. (B) Docking pose of AMP and the 13 compounds.

Table S2: Interaction free energy of the 10 ligands binding to the interface of PDBID 1Y64. The structures of the ligands are shown in Supporting Figure S13.The binding affinity between the compound and the more weakly bound protein is emphasized with bold font. The 3 compounds with the lowest max{$\Delta\Delta G_{RS}$,$\Delta\Delta G_{LS}$} are expected to be the most promising stabilizers and are highlighted in gray.

Ligand	$\Delta\Delta G_{RS}$ (kcal/mol)	$\Delta\Delta G_{LS}$ (kcal/mol)	$\lvert\Delta\Delta G_{RS}$ - $\Delta\Delta G_{LS}\rvert$(kcal/mol)	$\Delta\Delta G_{(RL)S}$ (kcal/mol)
1	**-14.37 ± 1.93**	-21.05 ± 2.67	6.68 ± 2.83	-44.04 ± 5.05
2	**-15.23 ± 2.31**	-20.31 ± 2.15	5.08 ± 3.06	-39.07 ± 3.37
3	-20.17 ± 3.41	**-18.7 ± 7.02**	6.11 ± 4.62	-45.67 ± 8.23
4	-20.59 ± 3.11	**-16.75 ± 1.88**	4.28 ± 3.1	-45.99 ± 3.92
5	**-19.63 ± 2.91**	-21.36 ± 3.92	4.66 ± 3.32	-46.68 ± 2.41
6	**-22.95 ± 3.68**	-21.33 ± 3.39	5.7 ± 2.98	-55.41 ± 3.49
7	**-20.45 ± 1.8**	-26.34 ± 5.44	5.9 ± 3.88	-57.03 ± 7.17
8	-22.84 ± 2.23	**-19.06 ± 2.57**	4.23 ± 3.43	-47.07 ± 2.46
9	-22.91 ± 2.73	**-20.97 ± 2.96**	4.05 ± 3.36	-53.1 ± 3.57
10	-26.74 ± 6.37	**-10.94 ± 2.53**	15.8 ± 8.64	-45.17 ± 5.68

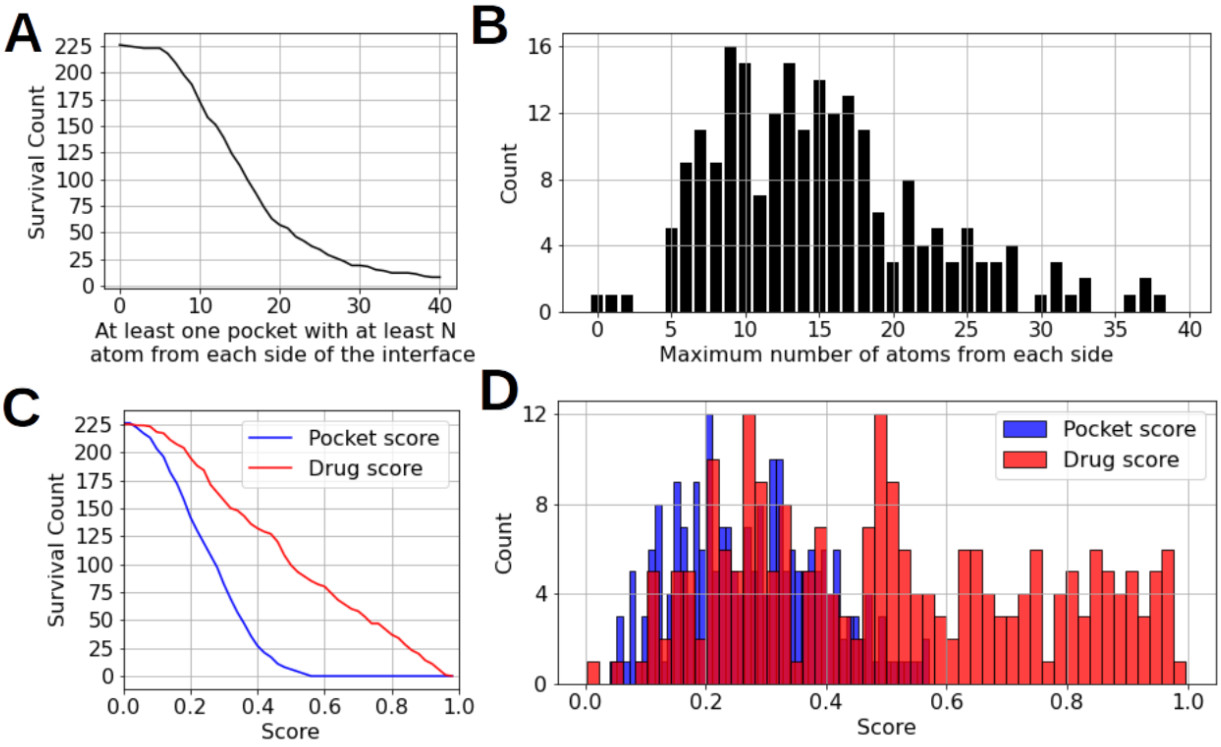

Figure S11: Pocket detection with Fpocket on the 226 PPI complexes from protein binding data set 5.0. (A) Survival curve of number of complexes with at least one pocket in contact with at least N atoms from each protein. (B) Histogram of the number of complexes with interface pocket maximally contacting N atom from each protein. (C) Survival curve of the number of complexes with maximum pocket score (blue) or drug score (red). (D) Histogram of the number of complexes with maximum pocket score (blue) or drug score (red). (C) and (D) consider only pockets contacting at least 5 atoms from each side of the protein-protein complex.

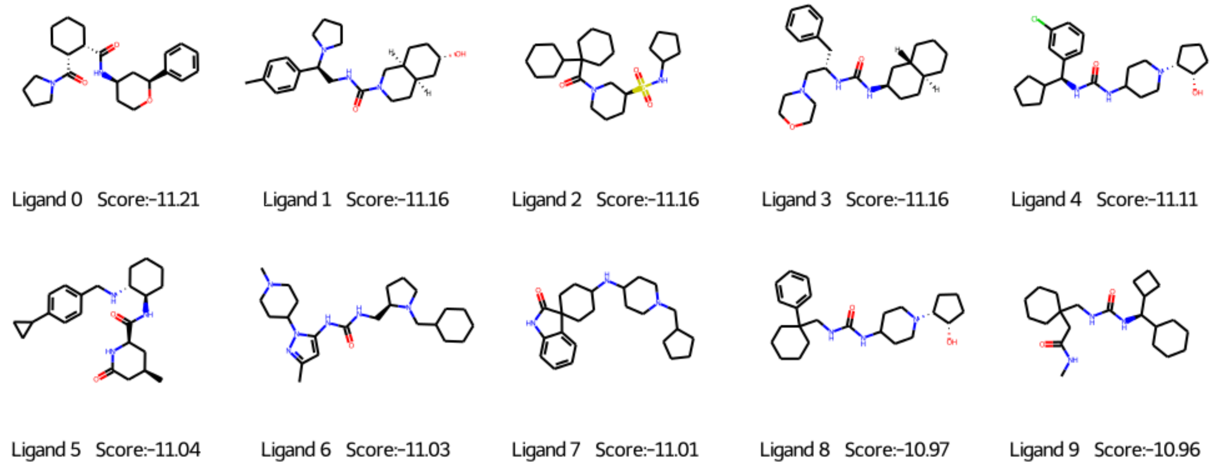

Figure S12: The best-docked 10 ligands to PDBID 1AK4 from virtual screening. The docking score is shown in a unit of kcal/mol.

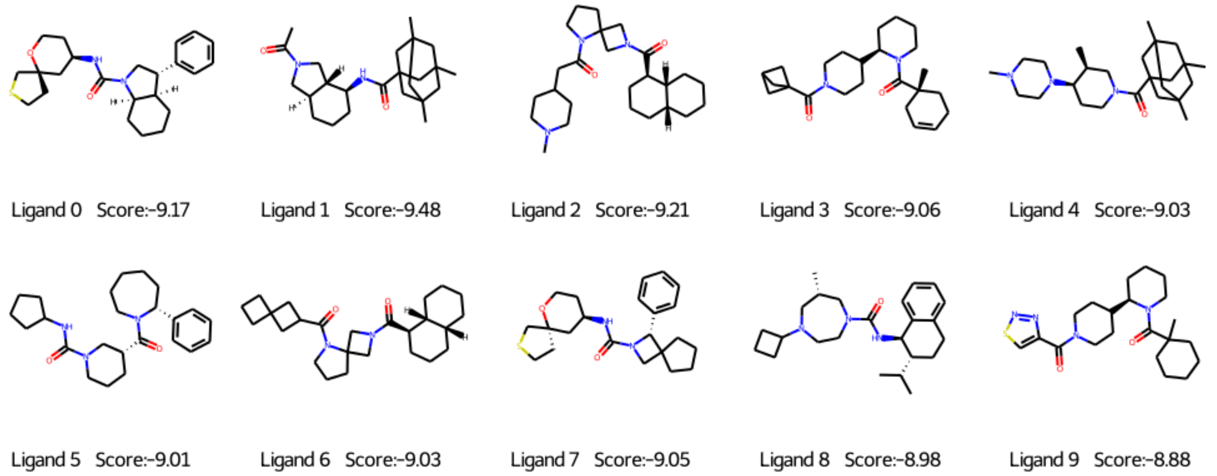

Figure S13: The best-docked 10 ligands to the PDBID 1Y64 interface from virtual screening. The docking score is shown in units of kcal/mol.

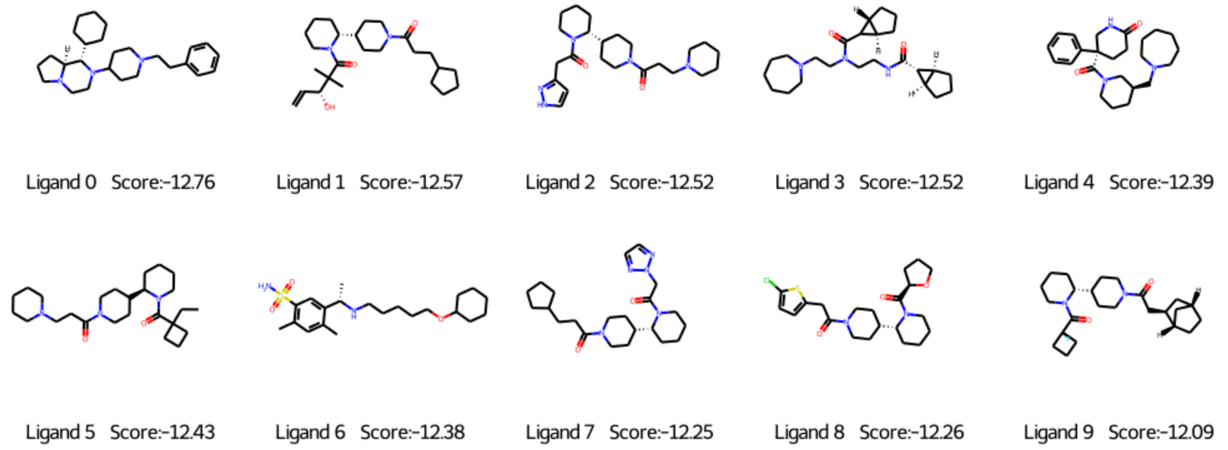

Figure S14: The best-docked 10 ligands found for target PDBID 2GAF from virtual screening. The docking score is shown in units of kcal/mol.

Table S3: Interaction free energy of the 10 ligands binding to the interface of PDBID 2GAF. The structures of the ligands are shown in Figure S14. The binding affinity between the compound and the more weakly bound protein is emphasized with bold font. The 3 compounds with the lowest max{$\Delta\Delta G_{RS}$,$\Delta\Delta G_{LS}$} are expected to be the most promising stabilizers and are highlighted in gray.

Ligand	$\Delta\Delta G_{RS}$ (kcal/mol)	$\Delta\Delta G_{LS}$ (kcal/mol)	$\|\Delta\Delta G_{RS} - \Delta\Delta G_{LS}\|$(kcal/mol)	$\Delta\Delta G_{(RL)S}$ (kcal/mol)
1	-42.45 ± 5.45	**-13.7 ± 6.18**	28.75 ± 7.68	-67.99 ± 11.8
2	-26.7 ± 4.5	**-17.63 ± 1.86**	9.07 ± 5.33	-51.99 ± 2.79
3	**-20.28 ± 4.76**	-27.79 ± 4.31	8.69 ± 6.96	-50.1 ± 1.49
4	-30.76 ± 3.7	**-19.89 ± 2.26**	10.89 ± 5.6	-56.84 ± 3.32
5	-29.24 ± 3.9	**-24.56 ± 3.42**	7.54 ± 2.65	-60.16 ± 6.78
6	-31.96 ± 3.02	**-22.51 ± 2.6**	9.45 ± 5.29	-60.84 ± 4.49
7	-31.59 ± 2.57	**-22.07 ± 3.36**	9.52 ± 5.4	-63.33 ± 4.81
8	-30.9 ± 5.24	**-20.59 ± 3.35**	10.31 ± 7.51	-56.93 ± 4.51
9	-27.68 ± 4.06	**-21.54 ± 1.82**	6.79 ± 4.44	-55.31 ± 4.56
10	-27.11 ± 4.78	**-21.79 ± 3.82**	7.78 ± 1.91	-57.1 ± 6.32

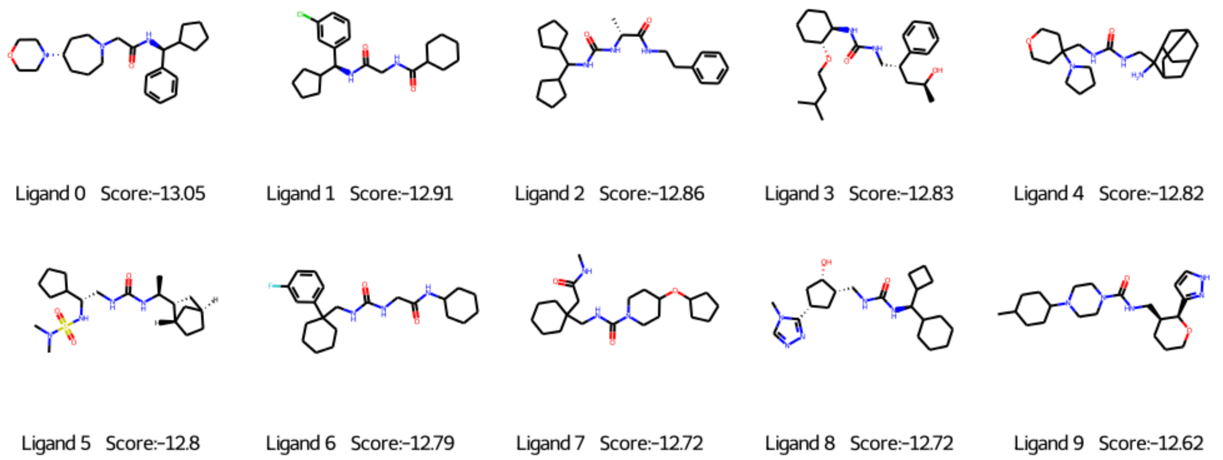

Figure S15: The best-docked 10 ligands for target PDBID 3H11 from virtual screening. The docking score is shown in units of kcal/mol.

Table S4: Interaction free energy of the 10 ligands binding to the interface of PDBID 3H11. The structures of the ligands are shown in Supporting Figure S15. The binding affinity between the compound and the more weakly bound protein is emphasized with bold font. The 3 compounds with the lowest max{$\Delta\Delta G_{RS}$,$\Delta\Delta G_{LS}$} are expected to be the most promising stabilizers and are highlighted in gray.

Ligand	$\Delta\Delta G_{RS}$ (kcal/mol)	$\Delta\Delta G_{LS}$ (kcal/mol)	$\|\Delta\Delta G_{RS}$ - $\Delta\Delta G_{LS}\|$(kcal/mol)	$\Delta\Delta G_{(RL)S}$ (kcal/mol)
1	**-20.95 ± 4.48**	-26.44 ± 2.24	5.49 ± 6.51	-50.62 ± 2.8
2	**-22.39 ± 1.68**	-25.49 ± 4.41	5.09 ± 3.27	-54.93 ± 4.55
3	**-14.98 ± 2.07**	-27.6 ± 2.4	12.62 ± 3.3	-48.89 ± 2.68
4	**-24.25 ± 2.9**	-29.86 ± 2.29	5.93 ± 3.34	-66.44 ± 5.65
5	**-16.45 ± 2.66**	-16.94 ± 3.69	4.22 ± 4.31	-42.73 ± 2.03
6	-40.25 ± 13.28	**-27.84 ± 2.7**	14.49 ± 11.44	-92.08 ± 14.15
7	**-18.57 ± 4.29**	-27.75 ± 0.94	9.17 ± 4.51	-55.27 ± 5.81
8	-22.57 ± 3.7	**-21.23 ± 1.49**	2.49 ± 1.72	-43.76 ± 4.42
9	**-22.88 ± 6.16**	-23.25 ± 4.85	10.24 ± 2.72	-54.42 ± 3.44
10	-20.72 ± 0.82	**-20.32 ± 3.81**	3.44 ± 2.46	-45.76 ± 4.39

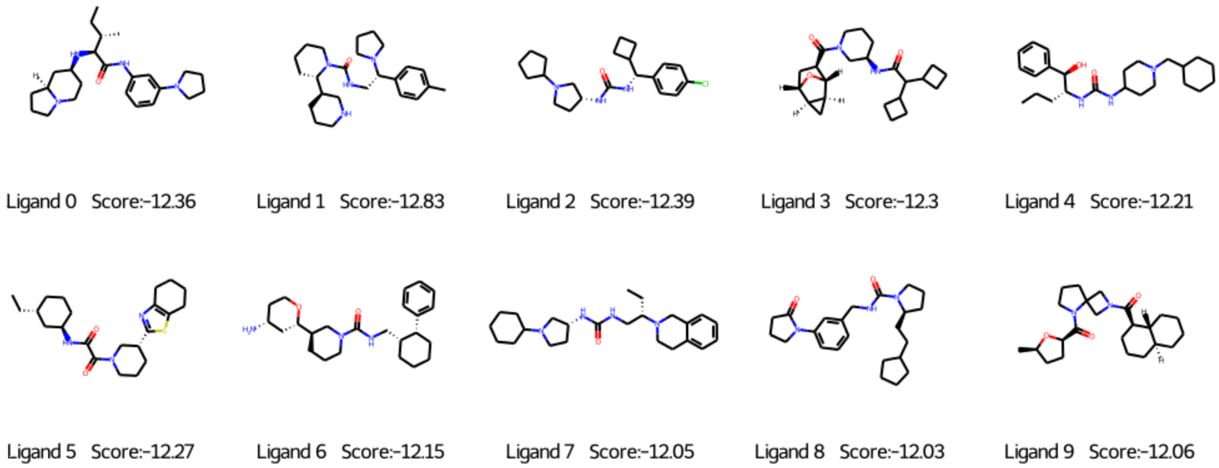

Figure S16: The best-docked 10 ligands to target PDBID 4FZA from virtual screening. The docking score is shown in units of kcal/mol.

Table S5: Interaction free energy of the 10 ligands binding to the interface of PDBID 4FZA. The structures of the ligands are shown in Supporting Figure S16. The binding affinity between the compound and the more weakly bound protein is emphasized with bold font. The 3 compounds with the lowest max{$\Delta\Delta G_{RS}$,$\Delta\Delta G_{LS}$} are expected to be the most promising stabilizers and are highlighted in gray.

Ligand	$\Delta\Delta G_{RS}$ (kcal/mol)	$\Delta\Delta G_{LS}$ (kcal/mol)	$\lvert\Delta\Delta G_{RS}$ - $\Delta\Delta G_{LS}\rvert$(kcal/mol)	$\Delta\Delta G_{(RL)S}$ (kcal/mol)
1	**-17.0 ± 3.98**	-28.95 ± 2.56	11.95 ± 5.2	-60.3 ± 6.6
2	**-23.23 ± 9.39**	-26.16 ± 2.28	8.18 ± 4.26	-53.9 ± 13.63
3	**-20.91 ± 1.48**	-23.36 ± 1.78	2.81 ± 1.65	-47.8 ± 2.73
4	**-16.59 ± 3.09**	-18.98 ± 3.79	2.41 ± 1.66	-40.9 ± 7.28
5	-24.63 ± 1.53	**-24.61 ± 0.96**	1.73 ± 1.1	-55.47 ± 1.2
6	-23.49 ± 4.4	**-22.02 ± 3.36**	5.49 ± 3.74	-55.59 ± 4.39
7	**-16.44 ± 3.16**	-30.32 ± 2.4	13.88 ± 3.24	-42.95 ± 7.69
8	**-25.84 ± 4.15**	-28.66 ± 2.8	3.63 ± 3.46	-60.94 ± 6.97
9	**-25.47 ± 2.02**	-26.72 ± 2.6	1.51 ± 1.31	-63.62 ± 6.45
10	-23.62 ± 1.98	**-21.83 ± 1.66**	2.41 ± 1.58	-57.46 ± 3.16

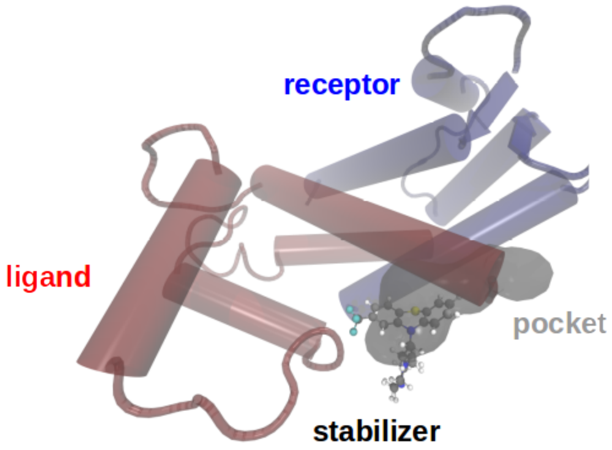

Figure S17: An illustration of the best-scored (pocket score) pocket detected from the C2 RL complex (PDBID: 3cga) using Fpocket with a ligand coverage fraction of 60%.

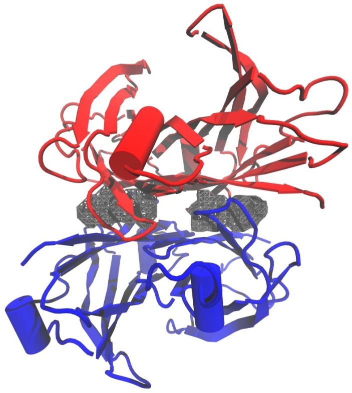

Figure S18: An illustration of the probes generated with 2Å around the ligand coordinates with TTR complex (PDBID 3tct).

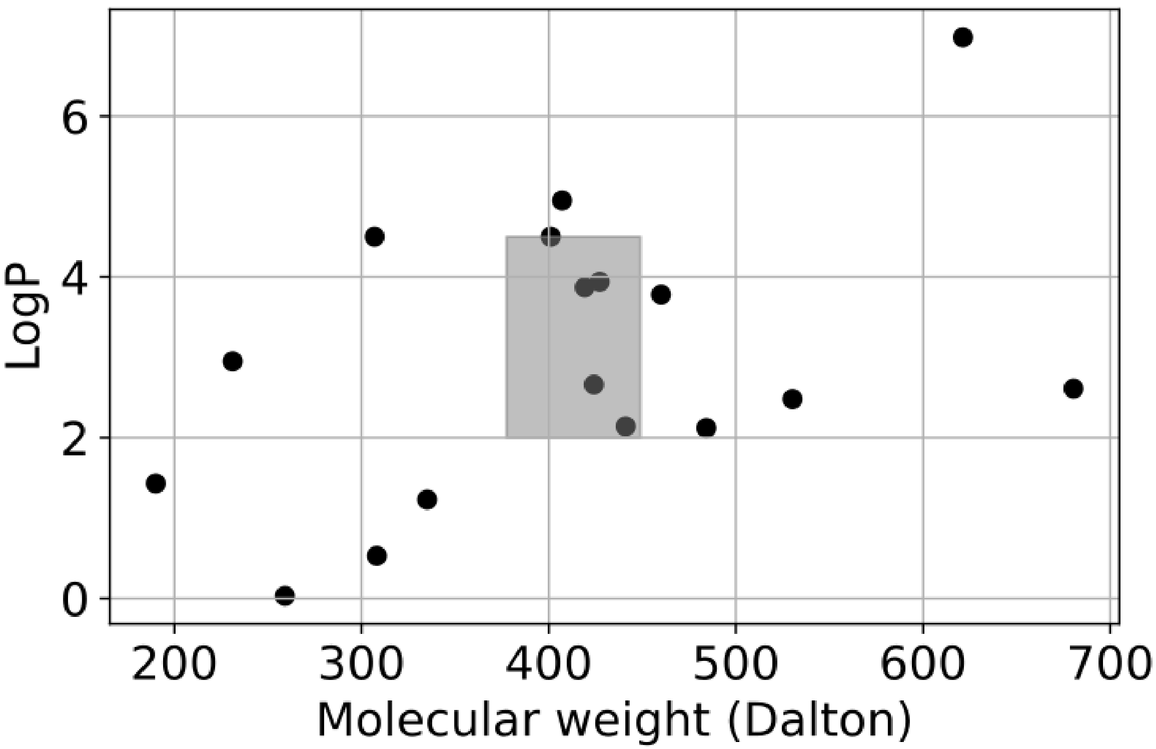

Figure S19: Population of the molecular weight and LogP of the stabilizers present in this study. The grey area indicates the range of molecular weight and LogP used to for virtual screening.
