# Supplementary material for: What Makes a Good Protein–Protein Interaction Stabilizer: Analysis and Application of the Dual-Binding Mechanism
Source: ACS Cent Sci. 2023 Apr 14;9(5):969–79. doi: 10.1021/acscentsci.3c00003 (PMC10214505; doi:10.1021/acscentsci.3c00003)
Supplement: Supplementary file 2 — oc3c00003_si_002.pdf [file oc3c00003_si_002.pdf]

oc-2023-00003e.R1

Name: Peer Review Information for "What makes a good protein-protein interaction stabilizer: Analysis and Application of the Dual-Binding Mechanism"

First Round of Reviewer Comments

Reviewer: 1

Comments to the Author

The manuscript ‘What makes a good protein-protein interaction stabilizer: Analysis and Application of the Dual-Binding Mechanism’ by Chen et al. is a very timely and well-executed bioinformatics analysis of the different aspects of the three-body-challenge of small-molecule PPI stabilizers. Basic features of the different classes of PPI stabilizers are discussed as well as practical applications like the in silico identification and ranking of stabilizers and the characterization of interface pockets that could accommodate PPI stabilizers in a set of 226 protein-protein complexes.

The latter two aspects are of utmost importance to the field. Identification of initial chemical matter for the development of small-molecule PPI stabilizers – molecular glues – is a substantial challenge and as far as I know has not been mastered computationally yet. Hence, the examples in this manuscript are of highest interest. It would have certainly been even more valuable if for the tested protein-protein complex entirely novel molecules would have been identified from scratch (by virtual screening with subsequent docking), however, I am aware that this would in itself be a stand-alone considerable work beyond the scope of the current manuscript.

The second aspect relates to the question how broadly applicable the concept of small-molecule PPI stabilization is across the entire human protein interactome. As a theoretical consideration of the need of less perfect and thus relatively weak regulatory protein-protein interactions, the resulting solvent-accessible rim-of-the interface regions should be enriched in ‘irregularities’, i.e. pocket, that could accommodate orthosteric PPI stabilizers. The results of this study seems to support this consideration and as such warrants a very optimistic view of the ‘stabilizer-ligandability’ of regulatory protein-protein complexes. The importance of this part of the current study cannot be overemphasized since it raises the incentive for elucidating the principles and practicalities of molecular glue drug discovery beyond the current examples, potentially to tens of thousands of (novel) drug discovery targets.

In conclusion, this is an excellent and very important study with wide implications for our fundamental understanding of the ‘three-body-challenge’ and the associated enormous value for drug discovery and ACS Central Sciences is the ideal journal for this study.

Reviewer: 2

Comments to the Author

The manuscript by Chen & Zacharias provides a quantitative analysis of the binding properties of ligands to protein-protein interfaces in protein complexes. Using MD and computational analysis of binding energy they analyze 18 characterized stabilizers of protein complexes. The authors find that

a similar interaction strength of the stabilizer to each protein partner is an important prerequisite for effective stabilization. The analysis is quantitative and technically well performed. Importantly the dataset of 15 PPIs, although not extensive, is varied in that it comprises a number of different scaffolds, increasing generalizability. Overall, using their model for PPI stabilizer ranking gave an improvement in ranking (compared to experiment) over other methods such as using the binding affinity to the ligand or total binding energy. The findings will have impact and will be of interest to a general audience. There are however concerns about some aspects of the experimental design and the data used by the authors to support some of their claims. Points which the authors may wish to consider are listed below in order of appearance (using the page numbers from the pdf for review).

Page: 7- I am concerned about the description of the interfaces with two stabilizers (Table 1). On inspection the two compounds in PDB 3m50 and 3m51 are adjacent but non-overlapping, those in 5j89 and 5j8o are partially overlapping and those in 5ad2 and 5ad3 are completely overlapping. So the ligand

binding surfaces being compared differ considerably in two of the cases. These differences should be described to the reader. Also, can the authors address the validity of the comparisons given these differences?

Page: 10- I am not clear that the data supports this statement for A3 "Indeed, comparison between the more potent and less potent stabilizers in set A shows that the weaker stabilizer-binding partner, namely […] has a stronger binding affinity to the more potent stabilizers than to the less potent stabilizers." To me the differences in A3 in delta delta GLS are within error (-27.95 kcal/mol and 28.7 kcal/mol). Unless I am mistaking the comparison being made?

Page: 14- The authors state "When the stabilizers are removed from the stabilizer-induced PPI complexes, the stabilizer-binding pockets remain stable in most complexes but the complexes adopt a

distorted interface (Figure 4D, Supplementary Figure S8, and Supplementary Figure S9)." I dont see how Figure 4D shows the distortion of the binding pocket as the ligand volume still fits within the pocket volume. Is there another way to compare the pockets before and after ligand removal? What would the pocket volume fluctuations be like when the simulation is run in the presence of the stabilizing ligand?

Page: 17- I do not believe the statement is well supported that "deltaGLS and deltaGRS of the ten ligands on each protein complex shows that, with few exceptions, most selected compounds are predicted to interact similarly with the receptor and ligand (Figure 5E)." Looking at that figure, there is not enough variation in the values to say that there is a correlation (fit to a line with slope of 1). Basically most all values in Figure 5E are approximately -20 kcal/mol, so indeed they are similar. Is there additional evidence to support the nature of the interaction with the receptor and ligand?

Grammar, typos

Page 2- "We employ molecular dynamics simulation and pocket detection techniques to investigate 18 known stabilizers and associated protein complexes." Should probably read "Herein, we employ ..."

Page: 3- The complex formation of proteins plays a fundamental role in the majority of biological

processes such as cell fate" should probably read "Protein-protein complex formation plays a ...."

Page: 4- Define “PP” as protein-protein at first instance

Page: 5- "We showed that the dual-binding mechanism can be useful to identify potential PPI stabilizers and from which we propose a protocol for PPI stabilizer discovery combining pocket probing, molecular docking, and MD simulation." This sentence needs to be restructured- there is a noun missing. Something like "...stabilizers and develop a model from which we propose a protocol....

Page: 9- “In addition, 7 other stabilizer-induced PPIs and 5 stabilizer-enhanced PPIs are also included in the study" this reads too redundantly - please remove the word also

Page 10- "It agrees with the theoretical model described in Eq.1, despite of weaker total stabilizer

interaction free energy" - remove the word "of"

Page: 12- "Such effect was observed in complexes A3, B6, and B7" should read "Such an effect was observed in complexes A3, B6, and B7.."

Author's Response to Peer Review Comments:

Garching, 28.2.2023

Dear Editor

Thank you for returning our manuscript entitled “What makes a good protein-protein interaction stabilizer: Analysis and Application of the Dual-Binding Mechanism " by Shu-Yu Chen and Martin Zacharias and the comments of the reviewers. In the following we like to comment on the concerns of the reviewers in a point-by-point response and indicate the changes and additions we have made to the manuscript. We include a version of the manuscript with all changes marked blue (deleted parts in red).

The key point that seems important to me includes the following aspects:

- deltaGLS and deltaGRS analyses in several cases (referee 2)

Author reply: See response to reviewer 2

Formatting Needs:

SI FILE: Please remove the "Abstract" heading from your Supporting Information file.

SI PAGINATION: SI pages must be numbered consecutively, starting with page S1.

SI TABLES: Please add the titles for the tables in your Supporting Information file above their respective tables, not below them like figures.

SI STATEMENT: Please provide a brief description of your Supporting Information (SI) in your SI paragraph.

ABSTRACT: Please shorten the abstract to 200 words or less.

SYNOPSIS: ACS Central Science requires a brief synopsis. The synopsis should be no more than 200 characters (including spaces) and should reasonably correlate with the Table of Contents (TOC) graphic. The synopsis is intended to explain the importance of the article to a broader readership across the sciences. Please place your synopsis in the manuscript file after the TOC graphic.

Author reply: We corrected all the above formatting needs and shortened the Abstract in the revised version of the manuscript. Changes are marked blue in the marked version. We also included a Synopsis statement (before the Introduction section).

Reviewer: 1

Recommendation: Publish in ACS Central Science without change.

Comments:

The manuscript 'What makes a good protein-protein interaction stabilizer: Analysis and Application of the Dual-Binding Mechanism' by Chen et al. is a very timely and well-executed bioinformatics analysis of the different aspects of the three-body-challenge of small-molecule PPI stabilizers. Basic features of the different classes of PPI stabilizers are discussed as well as practical applications like the in silico identification and ranking of stabilizers and the characterization of interface pockets that could accommodate PPI stabilizers in a set of 226 protein-protein complexes.

The latter two aspects are of utmost importance to the field. Identification of initial chemical matter for the development of small-molecule PPI stabilizers – molecular glues – is a substantial challenge and as far as I know has not been mastered computationally yet. Hence, the examples in this manuscript are of highest interest. It would have certainly been even more valuable if for the tested protein-protein complex entirely novel molecules would have been identified from scratch (by virtual screening with subsequent docking), however, I am aware that this would in itself be a stand-alone considerable work beyond the scope of the current manuscript.

The second aspect relates to the question how broadly applicable the concept of small-molecule PPI stabilization is across the entire human protein interactome. As a theoretical consideration of the need of less perfect and thus relatively weak regulatory protein-protein interactions, the resulting solvent-accessible rim-of-the interface regions should be enriched in 'irregularities', i.e. pocket, that could accommodate orthosteric PPI stabilizers. The results of this study seems to support this consideration and as such warrants a very optimistic view of the 'stabilizer-ligandability' of regulatory protein-protein complexes. The importance of this part of the current study cannot be overemphasized since it raises the incentive for elucidating the principles and practicalities of molecular glue drug discovery beyond the current examples, potentially to tens of thousands of (novel) drug discovery targets.

In conclusion, this is an excellent and very important study with wide implications for our fundamental understanding of the ‘three-body-challenge’ and the associated enormous value for drug discovery and ACS Central Sciences is the ideal journal for this study.

Author reply: We thank the reviewer for the encouraging comment.

Reviewer: 2

Recommendation: Reconsider after major revisions noted.

Comments:

The manuscript by Chen & Zacharias provides a quantitative analysis of the binding properties of ligands to protein-protein interfaces in protein complexes. Using MD and computational analysis of binding energy they analyze 18 characterized stabilizers of protein complexes. The authors find that a similar interaction strength of the stabilizer to each protein partner is an important prerequisite for effective stabilization. The analysis is quantitative and technically well performed. Importantly the dataset of 15 PPIs, although not extensive, is varied in that it comprises a number of different scaffolds, increasing generalizability. Overall, using their model for PPI stabilizer ranking gave an improvement in ranking (compared to experiment) over other methods such as using the binding affinity to the ligand or total binding energy. The findings will have impact and will be of interest to a general audience. There are however concerns about some aspects of the experimental design and the data used by the authors to support some of their claims. Points which the authors may wish to consider are listed below in order of appearance (using the page numbers from the pdf for review).

Author reply: We thank the reviewer for the encouraging comment.

1. Page: 7- I am concerned about the description of the interfaces with two stabilizers (Table 1). On inspection the two compounds in PDB 3m50 and 3m51 are adjacent but non-overlapping, those in 5j89 and 5j8o are partially overlapping and those in 5ad2 and 5ad3 are completely overlapping. So the ligand binding surfaces being compared differ considerably in two of the cases. These differences should be described to the reader. Also, can the authors address the validity of the comparisons given these differences?

Author reply: The reviewer is correct that in the two cases the interface surfaces differ. However, in order to evaluate these cases the analysis of the total contact surface (or buried surface) between stabilizer and protein partners is a valuable first approach. The calculated buried surface is a basic measure and related to the number of contacts between stabilizer and proteins. Hence, it is of interest to report that indeed for most of the effective stabilizers the buried (or contact) surface with respect to both protein partners is similar (reported in Figure 3A) despite the fact the type and structure of the surface differ. The physico-chemical differences of the contacts are considered later in the manuscript when comparing the mean interaction energies. We added an explanation on page 8 and also indicated the different binding modes that nevertheless resulted in similar protein-protein complex geometries.

2. Page: 10- I am not clear that the data supports this statement for A3 "Indeed, comparison between the more potent and less potent stabilizers in set A shows that the weaker stabilizer-binding partner, namely […] has a stronger binding affinity to the more potent stabilizers than to the less potent

stabilizers." To me the differences in A3 in delta delta GLS are within error (-27.95 kcal/mol and 28.7 kcal/mol). Unless I am mistaking the comparison being made?

Author reply: The reviewer compares in her/his statement the interaction of two different stabilizers (A3-a: compound 6 and A3-b: compound 3 as indicated in Table 1) with one protein partner (A3: BRD4 protein). Just by chance these compounds have similar calculated interaction energies with this partner. However, we are looking here at the calculated weaker interaction with one protein partner, in the A3-a case this is indeed the interaction with the ligand protein (-27.9 vs -29.6 kcal/mol with the receptor protein) whereas in A3-b the stabilizer interacts less with the receptor protein (-25.9 kcal/mol). Hence, it follows the claim that the magnitude of interaction with the weaker binding partner determines the stabilizer efficiency (in this case it is A3-a), which is experimentally also observed, pK_A3a=8.1 vs. pK_A3b=7.2). We modified the statement on page 9.

3. Page: 14- The authors state "When the stabilizers are removed from the stabilizer-induced PPI complexes, the stabilizer-binding pockets remain stable in most complexes but the complexes adopt a distorted interface (Figure 4D, Supplementary Figure S8, and Supplementary Figure S9)." I don't see how Figure 4D shows the distortion of the binding pocket as the ligand volume still fits within the pocket volume. Is there another way to compare the pockets before and after ligand removal? What would the pocket volume fluctuations be like when the simulation is run in the presence of the stabilizing ligand?

Author reply: After re-inspection of the original SI Figure S8 and Figure S9 we agree that the presentation is very noisy and the main effects are not well visible. In the revised version, we revised the Figures plotting a sliding window average (averaging over a time window of 1ns). This illustrates much more clearly the trend in the volume change of the binding pockets (in the absence of the stabilizer). For consistency, we also updated Figures that show interface RMSD (iRMSD, Figure 3C and SI Figure S3-S5) using the same sliding average procedure. The new Figures give a much clearer view on the significant decrease in volume of a stabilizer-binding pocket as observed in the stabilizer-induced RL complexes such as A3 (blue and purple), B1 (orange), B6 (blue and green), B5 (orange), and B7 (orange) when the stabilizers are removed. As suggested by the reviewer, we include the pocket size distribution in the simulations of the RLS ternary complexes in Figure 4D and compare it directly to the simulations after removal of the stabilizer. In case of the RLS complexes the sampled volume is always larger than the volume of the ligand whereas sampling of states with pocket volumes below the stabilizer volume is evident in several cases after removal of the stabilizer. We added a paragraph to better explain what is meant by the 'distorted interface'.

4. Page: 17- I do not believe the statement is well supported that "deltaGLS and deltaGRS of the ten ligands on each protein complex shows that, with few exceptions, most selected compounds are predicted to interact similarly with the receptor and ligand (Figure 5E)." Looking at that figure, there is not enough variation in the values to say that there is a correlation (fit to a line with slope of 1). Basically most all values in Figure 5E are approximately -20 kcal/mol, so indeed they are similar. Is there additional evidence to support the nature of the interaction with the receptor and ligand?

Author reply: We agree with the reviewer on the weakness of Figure 5E and revised the Figure. We want to emphasis, however, that the diagonal line in Figure 5E is not a fit curve and our intention was not to show the correlation between ΔΔGLS and ΔΔGRS but to show that most data points are not far off with respect to the diagonal line, which means that the calculated ΔΔGLS and ΔΔGRS are not too different from each other. It is important to note and one should keep here in mind that depending on the

compound library and the considered system (and depending also on the docking procedure) not in all cases the ten best identified compounds fulfill the dual-binding mode criterion perfectly and will, hence, give a perfect stabilizer.

We agree that the diagonal line in Figure 5E can be misleading and thus adopted a new more useful plot for Figure 5E instead. Since we want to emphasize the importance of the weaker interacting partner, indicated by max{ΔΔGLS,ΔΔGRS}, and the similarity in interaction energy, as indicated by |ΔΔGLS - ΔΔGRS |, the new plot is generated with respect to these parameters. The plot shows that the compounds at the lower-left corner are expected to be the most promising stabilizers because they have a very favorable predicted interaction even with the weaker partner and a small difference in interaction with both protein partners. We adapted the text accordingly (marked blue in the marked version).

To be consistent with the main part and avoid confusion we also adapted the SI Tables S1-S5 and list the calculated absolute interaction energy difference |ΔΔGLS-ΔΔGRS| which can be more easily compared with the mathematical model shown in Figure 1D and the absolute binding energy difference in PPI examples in Table 2.

5. Grammar, typos

Page 2- "We employ molecular dynamics simulation and pocket detection techniques to investigate 18 known stabilizers and associated protein complexes." Should probably read "Herein, we employ ..."

Page: 3- The complex formation of proteins plays a fundamental role in the majority of biological processes such as cell fate" should probably read "Protein-protein complex formation plays a ...."

Page: 4- Define “PP” as protein-protein at first instance

Page: 5- "We showed that the dual-binding mechanism can be useful to identify potential PPI stabilizers and from which we propose a protocol for PPI stabilizer discovery combining pocket probing, molecular docking, and MD simulation." This sentence needs to be restructured- there is a noun missing. Something like "...stabilizers and develop a model from which we propose a protocol....

Page: 9- “In addition, 7 other stabilizer-induced PPIs and 5 stabilizer-enhanced PPIs are also included in the study" this reads too redundantly - please remove the word also

Page 10- "It agrees with the theoretical model described in Eq.1, despite of weaker total stabilizer interaction free energy" - remove the word "of"

Page: 12- "Such effect was observed in complexes A3, B6, and B7" should read "Such an effect was observed in complexes A3, B6, and B7.."

Author reply: We thank the reviewer for indicating the grammar and spelling errors. We corrected all errors and carefully re-checked the manuscript.

Finally, we like to thank the reviewers and you for the comments that, we believe, improved our manuscript and hope that with the additions and changes we have made to the manuscript it is now acceptable for publication in the ACS Central Science.

Yours sincerely,

Martin Zacharias
